# The female gametophyte: an emerging model for cell type-specific systems biology in plant development

**DOI:** 10.3389/fpls.2015.00907

**Published:** 2015-11-03

**Authors:** Marc W. Schmid, Anja Schmidt, Ueli Grossniklaus

**Affiliations:** Department of Plant & Microbial Biology and Zurich-Basel Plant Science Center, University of ZurichZurich, Switzerland

**Keywords:** developmental systems biology, model systems, single cell type isolation, gametophyte, transcriptomics

## Abstract

Systems biology, a holistic approach describing a system emerging from the interactions of its molecular components, critically depends on accurate qualitative determination and quantitative measurements of these components. Development and improvement of large-scale profiling methods (“omics”) now facilitates comprehensive measurements of many relevant molecules. For multicellular organisms, such as animals, fungi, algae, and plants, the complexity of the system is augmented by the presence of specialized cell types and organs, and a complex interplay within and between them. Cell type-specific analyses are therefore crucial for the understanding of developmental processes and environmental responses. This review first gives an overview of current methods used for large-scale profiling of specific cell types exemplified by recent advances in plant biology. The focus then lies on suitable model systems to study plant development and cell type specification. We introduce the female gametophyte of flowering plants as an ideal model to study fundamental developmental processes. Moreover, the female reproductive lineage is of importance for the emergence of evolutionary novelties such as an unequal parental contribution to the tissue nurturing the embryo or the clonal production of seeds by asexual reproduction (apomixis). Understanding these processes is not only interesting from a developmental or evolutionary perspective, but bears great potential for further crop improvement and the simplification of breeding efforts. We finally highlight novel methods, which are already available or which will likely soon facilitate large-scale profiling of the specific cell types of the female gametophyte in both model and non-model species. We conclude that it may take only few years until an evolutionary systems biology approach toward female gametogenesis may decipher some of its biologically most interesting and economically most valuable processes.

## 1. Systems biology: an integrated approach to model biological processes with large-scale data

Since the foundation of the Institute for Systems Biology in the year 2000 and the formal definition of systems biology at the beginning of the twenty-first century (Ideker et al., 2001; Kitano, 2002), it has been a steadily growing field of research. As an integrative approach, systems biology is markedly different from the reductionistic approach generally used in molecular biology and genetics. Powered by the central dogma of biology, where a gene is transcribed to mRNA, which is then translated into proteins, molecular biology and genetics have successfully identified genes, their functions, and the processes they are involved in. However, the implicit link of a gene to a certain function or a phenotype is an oversimplification of the underlying process. It thus frequently misses important interactions with other cellular or environmental factors (e.g., responses to environmental conditions like a temperature-dependent phenotype of a mutant). In contrast, systems biology may be described as an attempt to quantitatively and/or qualitatively describe and understand the global behavior of a biological entity, emerging from the interactions between its molecular components. Such a comprehensive understanding would allow the prediction and modeling of the biological entity, its precise control, and ultimately the targeted manipulation of a complex biological system (reviewed in Kitano, 2002; Yuan et al., 2008; Fukushima et al., 2009; Chuang et al., 2010; Katari et al., 2010; Weckwerth, 2011).

Systems biology comprises and integrates experimental studies and large-scale data sets derived from high-throughput technologies (omics), such as transcriptomics (RNA profiling), proteomics (analysis of proteins), and metabolomics (profiling of metabolites). However, also epigenetic regulatory processes based on the modification of chromatin components or DNA (epigenomics), the translation of mRNAs to proteins (translatomics), complex formation of proteins with proteins or nucleic acids (interactomics), the investigation of protein modifications, e.g., phosphorylation important for the regulation of their activity (phospho-proteomics), and the transport of ions or metabolites (fluxomics) need to be taken into account to achieve a full picture of the dynamic processes of a cell or organism (reviewed by Sheth and Thaker, 2014). One of the most crucial aspects for systems biology approaches is the comprehensiveness of the omics data (Kitano, 2002). For a given method this includes the number of items that can be measured at once (e.g., transcripts with transcriptomics). For the entire system, it is then important whether the relevant items (e.g., enzymes and metabolites) or processes (e.g., posttranslational modifications) can be accurately measured with a combination of certain methods. An additional level of complexity may be imposed by the requirement of a high spatial and/or temporal resolution. For a single, isolated cell this can refer to specific organelles, subcellular compartments, certain domains of the plasma membrane, and the stage of the cell-cycle. For an unicellular organism like yeast, this may be augmented by studying the cell-to-cell variability within the population (Pelkmans, 2012). In multicellular organisms, each cell (type) has a specific function and position within an organ. Its role and differentiation status may be influenced by local signals as well as systemic signals originating from other organs (e.g., hormones). In addition, the temporal coordinate expands to developmental stages of the organs or the life span of the organism.

Consequently, a complete understanding at the systems level requires highly resolved, quantitative spatio-temporal data on the individual components and their interactions, and the integration of the data into models. On one hand, integration of these data with computational methods can aid to characterize previously unknown components (e.g., genes) of a system, as exemplified for yeast (Brown et al., 2006). Alternatively, the data may be used in a mathematical model describing the system and allowing the prediction of a system's behavior and the formulation of hypotheses (Süel et al., 2007). Finally, the integration of omics data, the formulation of mathematical models, the generation of hypotheses, and the experiments are interlinked and benefit from each other. A possible extension of systems biology is the use of interspecies comparisons to, for example, elucidate the extent to which genotypic variation translates into phenotypic differences (Konstantinidis et al., 2009). Even broader, evolutionary systems biology may be recognized as an approach to describe and understand how biological systems are shaped by evolution and are steering it at the same time (reviewed in Soyer, 2012).

Prior to the understanding of a complex organism composed of many different cell and tissue types, investigations of distinct cell types can lead to an understanding of basic processes governing cellular specification, differentiation, and metabolism. To date, yeast (*S. cerevisiae*) is a widely used model system appreciated as the currently best understood cell (Boone, 2014). While evolutionary only distantly related, pathways in yeast have shown to have considerable similarities to the ones in plants, animals, and humans (Ideker et al., 2001). In addition, yeast serves for the production of food and pharmaceuticals. Due to its simplicity and its importance for biotechnology and biomedical research, yeast has shaped modern molecular biology to a great extent. Indeed, it has been a pioneering organism in systems biology (reviewed in Bostein and Fink, 2011; Österlund et al., 2012; Boone, 2014), starting from gene expression and regulatory networks discovered during early transcriptome studies and their integration with other genome-wide data, over genetic interaction networks obtained by crossing thousands of mutant strains (Costanzo et al., 2010) and modeling of gene expression as a Quantitative Trait Locus (eQTL, Brem et al., 2002), to genome-wide metabolic models. However, given the unicellularity of yeast, it can hardly serve as a developmental model for complex multicellular animals and even less so for plants. In plants, systems biology is less advanced for several reasons, including the higher complexity of most plant genomes, large gene families, the multitude of primary and secondary metabolites, and the lack of suitable *in vitro* systems or cell lines for most plant tissues. Most efforts in plant research thus require *in vivo* experiments, making the procedures generally more difficult and less suitable to high-throughput approaches. As a consequence, data generation can be a severely limiting factor for plant systems biology. On the other hand, the results are of high relevance for the process under investigation.

**Figure 1:**
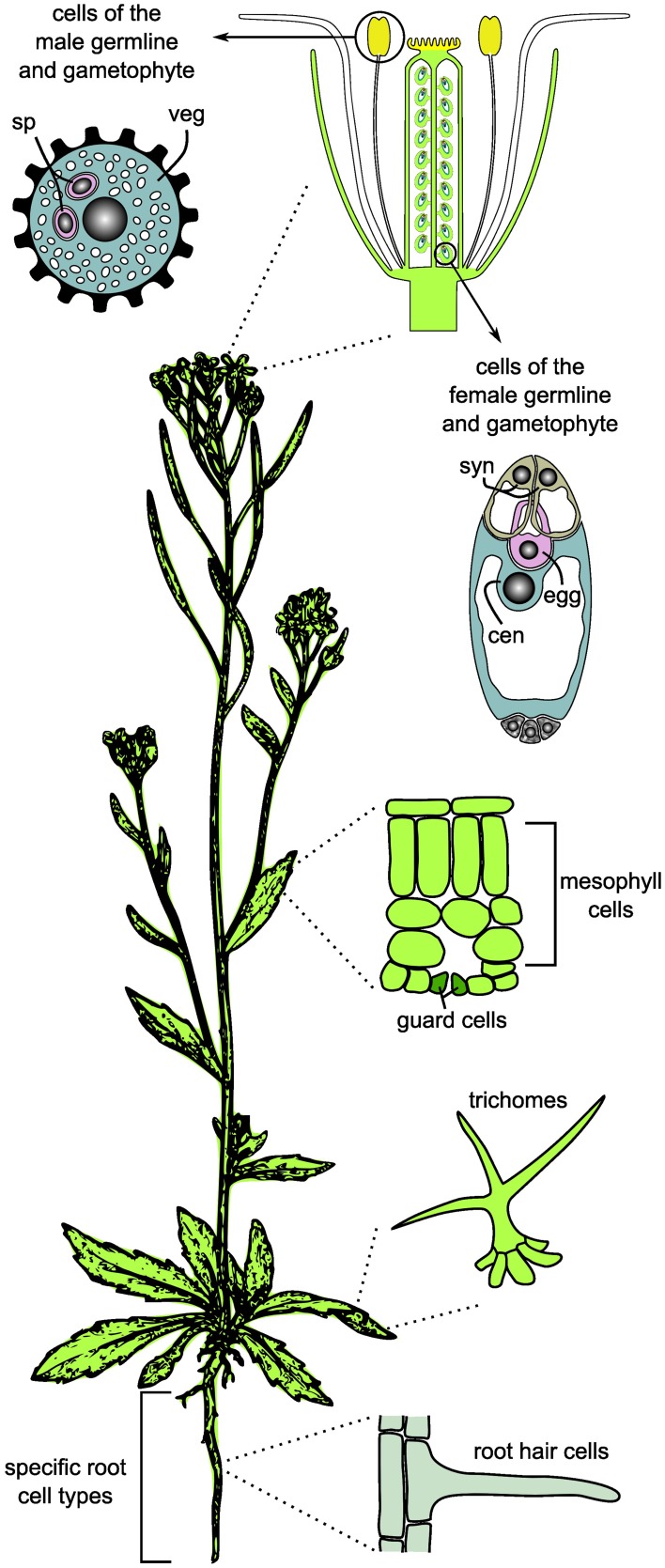
**Cell and tissue types frequently used for cell type-specific systems biology and omics studies in plants**. For the germlines, only the mature gametophytes are shown. sp, sperm cell; veg, vegetative cell; syn, synergids; cen, central cell; egg, egg cell.

Apart from the above mentioned obstacles, substantial progress in the analysis of specific cell types in plants has been made over the last decade. Facilitated by advances in high-throughput profiling technologies and methods for the isolation of individual cell types, recent studied focussed on the analysis of specific cell types or even single cells (Figure 1). To investigate cell type-specific processes in higher plants, root hairs and trichomes have been used as models, both for their physiological importance and their accessibility at the epidermal surface (for details see below; Ishida et al., 2008; Brechenmacher et al., 2009, 2010; Dai et al., 2010; Libault et al., 2010a,b; Schilmiller et al., 2010; Nestler et al., 2011; Van Cutsem et al., 2011; Dai and Chen, 2012; Rogers et al., 2012; Tissier, 2012; Qiao and Libault, 2013). In addition, starting with only a few examples at the beginning of the twenty-first century (Kehr, 2001), cell type-specific transcriptional profiling has become a robust and frequently used method. In the model plant *Arabidopsis thaliana*, novel insights into plant development and cellular responses to environmental stimuli were for example gained through studies on individual cell types of the root, root hairs, trichomes, and guard cells, and by transcriptional profiling during male and female gametogenesis (reviewed in Taylor-Teeples et al., 2011; Schmidt et al., 2012; Wuest et al., 2013). These examples clearly illustrate the importance of cell type-specific investigations for a detailed understanding of differentiation processes and environmental responses of distinct cell types. However, depending on the cell type under investigation, the currently available methods for cell isolation may be challenging, time-consuming, or limited to a subset of omics approaches (e.g., Laser-Assisted Microdissection (LAM) of rare cell types, Wuest et al., 2013). While studies focusing on specific cell types, which can be isolated in quantities high enough for the full set of omics approaches, can serve as initial models for cell type-specific systems biology in plants (Libault et al., 2010a), the ultimate goal must be that the full set of methods can be applied to any cell type of interest.

## 2. Methods for the acquisition of large-scale quantitative data from specific cell types

Large-scale profiling of distinct cell types critically depends on the possibility to isolate these cells in sufficient purity and quantity, as well as the sensitivity and accuracy of the profiling methods. Despite the rapid improvements of established and novel tools for systems biology, the demand for fast and easily applicable methodologies for cell type-specific analyses is not yet satisfied. Further challenges are associated with the requirement for normalization and integration of different data types, and the increasing demand for platforms allowing storage and sharing of the rapidly growing amount of large-scale datasets (reviewed by Chuang et al., 2010; Katari et al., 2010; Gomez-Cabrero et al., 2014; Sheth and Thaker, 2014). In brief, three steps are of great importance for cell type-specific systems biology: (i) isolation and purification of the specific cell type, (ii) profiling of the selected molecular compounds, and (iii) data analysis, integration, storage, and sharing. In the following sections, we will present current methods to acquire large-scale quantitative data required for systems biology. We will focus on methods allowing genome-wide cell type-specific analyses and present representative examples. For a discussion on the computational challenges in systems biology, the reader is referred to several recent reviews (Ahrens et al., 2007; Yuan et al., 2008; Fukushima et al., 2009; Chuang et al., 2010; Katari et al., 2010; Liberman et al., 2012; Fukushima et al., 2014; Gomez-Cabrero et al., 2014; Robinson et al., 2014).

### 2.1. Methods for the isolation of specific cell types

A few cell types in plants are exposed on the surfaces of tissues and can be collected by abrasion or mechanical detachment. Depending on the species, relatively simple mechanical isolation procedures for trichomes and root hairs enabled a large spectrum of methods. Mechanical isolation of trichomes allowed transcriptomics and metabolomics in various species (for an integrated database see Dai et al., 2010) and proteomics (Schilmiller et al., 2010; Van Cutsem et al., 2011). Another example for an exposed cell type are root hairs, for which relatively simple isolation procedures facilitated transcriptomics (Libault et al., 2010b), proteomics (Brechenmacher et al., 2009; Nestler et al., 2011), and metabolomics (Brechenmacher et al., 2010). Certain other cell types can be isolated by tissue disruption, followed by centrifugation-based methods or manual isolation of the dissociated cells under a microscope using a micropipette (eventually with a marker for the cell type of interest). Examples include specific cell types from the male or female reproductive lineages, plant mesophyll cells, and guard cells (reviewed by Dai and Chen, 2012; Schmidt et al., 2012; Wuest et al., 2013). Proteomic profiling has, for example, been performed on *Brassica napus* guard cells and mesophyll cells that could be purified as protoplasts (Zhu et al., 2009).

However, for most cell types these methods are not applicable. Several methods for the isolation of specific cell types embedded in differentiated tissues have been established. Fluorescent Activated Cell Sorting (FACS) can be used to sort fluorescent cells based on their light scattering characteristics and fluorescence (reviewed by Hu et al., 2011). This method allowed high resolution transcriptional profiling of different cell types in the *Arabidopsis* root, and, more recently, proteomics (Petricka et al., 2012) and metabolite mapping of selected root cell and tissue types (Brady et al., 2007; reviewed by Benfey, 2012; Moussaieff et al., 2013). Similarly, Fluorescence-Activated Nuclei Sorting (FANS) has been established and, for example, used to isolate endosperm nuclei for profiling of RNA activity or epigenetic modifications (Weinhofer et al., 2010; Weinhofer and Köhler, 2014). Despite the great potential of FACS/FANS for plant cell type-specific systems biology, both approaches have certain limitations: They can only be applied if transgenic lines carrying cell type-specific fluorescent markers can be established, and they are thus not suitable for most non-model species. In addition, depending on the tissue type, longer enzymatic incubations are required to digest the cell walls and to release the protoplasted cells prior to sorting (Evrard et al., 2012). Consequently, changes in, for example, the transcriptome or metabolome cannot be fully excluded. Alternatively, the INTACT method (Isolation of Nuclei TAgged in specific Cell Types) allows the isolation of nuclei expressing a biotinylated nuclear envelope protein by affinity purification with streptavidin-coated beads (Deal and Henikoff, 2011). This method is suitable to study epigenetic modifications (DNA methylation of histone modifications) and to profile the RNA within the nucleus. To study actively translated mRNAs bound to ribosomes (translatome), small epitope tags can be fused to a ribosomal protein to allow immunopurification of the ribosomes containing the mRNAs with a method named TRAP (Translating Ribosome Affinity Purification; reviewed in Bailey-Serres, 2013). Alternatively, RNAs binding to RNA binding proteins involved in the formation of ribonucleoprotein (RNP) complexes can be profiled by immunoprecipitation of an epitope-tagged protein (RNP ImmunoPurification, RIP; Bailey-Serres, 2013). It has to be noted that the analyses of transcriptome and translatome abundance will not give the same results, because not all mRNAs present in a cell are actively translated at a given time point. In this respect, profiling of mRNAs bound to ribosomes gives complementary results to transcriptome profiling as the readouts are closer to the synthesis of proteins (Bailey-Serres, 2013). Similar to FACS and FANS, also INTACT, TRAP, and cell type-specific RIP require the use of transgenic lines and pre-existing knowledge about cell type-specific promoters or markers.

An alternative method not requiring any molecular knowledge is LAM (Kerk et al., 2003). Plant tissues are thereby typically fixed and embedded in paraffin wax (reviewed in Schmidt et al., 2012; Wuest et al., 2013) or resin (Tucker et al., 2012; Okada et al., 2013). Thin sections of the tissues (typically between 6 and 10 μm) are subsequently mounted on metal framed plastic slides and used to isolate the cell types of interest after resolving the wax or resin and drying the tissues on the slides (Okada et al., 2013; Wuest and Grossniklaus, 2014). Alternatively, the tissue may also be embedded in optimal cutting temperature compound for cryosectioning, followed by on-slide tissue dehydration and LAM (Kelliher and Walbot, 2012, 2014). The main constraint of LAM is that harvesting sufficient material for downstream omics methods can be very time-consuming. Furthermore, the suitability for single cell isolation depends on the optical resolution in sectioned tissues and the recognizability of the cell type of interest. In addition, the physical properties of the laser beam of the instrument used can impose limitations on which cell types can be isolated (Schmidt et al., 2012). Thus, the time required for collecting enough material for one sample is largely dependent on the cell type of interest. So far, the applications of LAM for cell type-specific omics have been restricted to transcriptional profiling, e.g., to study cell type-specification in the female reproductive lineage in *Arabidopsis thaliana, Boechera gunnisoniana*, and *Hieracium praealtum* (Wuest et al., 2010; Schmidt et al., 2011, 2014; Schmid et al., 2012; Okada et al., 2013). However, other applications, such as genome wide profiling of DNA methylation, are likely feasible (see below).

### 2.2. Methods for data acquisition

#### 2.2.1. Transcriptomics

Transcriptome profiling encompasses the identification and quantification of all expressed RNA transcripts at a given time point (mRNA, tRNA, microRNA). However, due to the frequent use of oligo-dT priming during cDNA synthesis or the hybridization to microarrays covering only coding regions of the genome, many studies are restricted to mRNAs or a subset of mRNAs. Several types of microarrays were produced and extensively used for the analyses of gene expression in different plant species, including the model plant *Arabidopsis thaliana* and different important crop species like maize, rice, and barley (reviewed in Sheth and Thaker, 2014). The Affymetrix ATH1 GeneCHIP (www.affymetrix.com), the most popular microarray for *Arabidopsis* has for example been used to profile a large variety of different tissue types (e.g., Schmid et al., 2005), specific cell types of the root isolated through FACS (Birnbaum et al., 2003), and specific cell types of the male and female reproductive lineages (reviewed in Schmidt et al., 2012). In addition to well established tools for data analysis, the wealth of publicly available datasets generated on the same platform makes commonly used microarrays a very valuable tool for systems biology (Katari et al., 2010).

Apart from microarrays, several platforms for Next Generation Sequencing (NGS) have been developed over the last years and are now routinely used for transcriptional profiling (RNA-Seq; see Mardis, 2013, for a review on NGS platforms). RNA-Seq has several advantages as compared to the use of microarrays, including a higher dynamic range, higher sensitivity, and whole-genome coverage allowing the identification of previously unknown transcripts and splice variants (reviewed in Schmidt et al., 2012). A major advantage is the applicability to non-model species, either through *de novo* assembly of the short reads into transcripts or by the use of a reference transcriptome either produced separately or taken from a public database (e.g., the ongoing effort to sequence 1000 plant transcriptomes, www.onekp.com). Examples for such an approach are the central cells of *Arabidopsis thaliana*, and cells of the female reproductive lineage in *Hieracium praealtum* and *Boechera gunnisoniana* (Schmid et al., 2012; Okada et al., 2013; Schmidt et al., 2014). Several tools for RNA-Seq data analysis are available (see Sheth and Thaker, 2014, for a selection of software tools, and Schmid and Grossniklaus, 2015, for Rcount, a count tool addressing the problem of reads aligning at multiple locations in the genome, or reads aligning at positions where two or more genes overlap). Current challenges are the increasing demand for standardized annotations of datasets and the development of computational methods allowing the integration of data from different studies using different methods and platforms. In the future, the integration of data from different species will be of great value for plant systems biology, allowing researchers to gain insights into conserved common regulatory mechanisms, environmental adaptations, and evolutionary changes.

#### 2.2.2. Proteomics

In addition to the analysis of gene expression and actively translated mRNAs, the investigation of proteins and protein modifications (e.g., phospho-proteomics and glyco-proteomics) add additional levels of complexity. From a systems biology perspective the aim is the combination of cell type-specific proteomics with transcriptomics and metabolomics to elucidate and model regulatory networks (reviewed in Dai and Chen, 2012). In the beginning of proteomics, 2D gel electrophoresis was frequently used for separation of the proteins in a sample and to identify spots representing proteins differentially occurring in two samples (reviewed by Schulze and Usadel, 2010). However, the protein or protein mixture in one spot could only be identified by excising the spot and analysis using Mass Spectrometry (MS). To date, proteomics largely depends on the use of various MS methods in combination with different protein separation procedures. Typically, proteins are first digested with trypsin and subsequently either analyzed directly by MS or first separated by chromatography before MS. MS methods have greatly improved with the development of soft ionization methods like ElectroSpray Ionization (ESI) in solution (typically aqueous or organic solvents) or Matrix Assisted Laser Desorption Ionization (MALDI, Hollenbeck et al., 1999; Schulze and Usadel, 2010). By both methods, intact gas phase ions are generated that are introduced into mass analyzers and sorted depending on their mass-to-charge ratio, e.g., using their Time-Of-Flight (TOF, Hollenbeck et al., 1999; for a recent summary of mass analyzers see Lee et al., 2012; for a description of Orbitrap mass analyzers see Perry et al., 2008). However, detection based on peptide mass-to-charge ratios is largely qualitative and can only be used for quantification in two or more samples acquired under standardized conditions (Schulze and Usadel, 2010). Thus, stable isotope or chemical labeling is frequently applied for quantification in proteomic methods (reviewed in Schulze and Usadel, 2010). While software and algorithms for protein identification are well established, quantitative analysis remains more challenging (Schulze and Usadel, 2010; Sheth and Thaker, 2014, see Sakata and Komatsu, 2014, for a recent survey on proteomics repositories and databases).

To date, only a restricted number of plant cell types have been profiled in a cell type-specific manner by proteomics, including guard cells, mesophyll cells, trichomes, root hair cells, leaf epidermal cells, lily and rice sperm cells, different stages of pollen development in tobacco, *Arabidopsis*, and tomato, and rice egg cells (Brechenmacher et al., 2009; Grobei et al., 2009; Abiko et al., 2013; Chaturvedi et al., 2013; Ischebeck et al., 2014, and reviewed by Dai and Chen, 2012; Wuest et al., 2013). As compared to transcriptomics approaches, a larger amount of starting material is required. For example, approximately 40 μg of protein were isolated to study the proteome during tobacco pollen development (Ischebeck et al., 2014). In addition, the amount of proteins detected is typically in the range of 10–30% of the transcripts identified from the same cell or tissue type, as exemplified by a study on *Arabidopsis* pollen, in which 3599 proteins as compared to 11,150 expressed genes were reported (Grobei et al., 2009). This quantitative difference largely reflects the difference in the sensitivity of the methods and likely only to a smaller extent meaningful biological differences. Nevertheless, as only a few proteins have been identified in previous studies, e.g., from maize egg cells, these data reflect a great improvement (Okamoto et al., 2004), and a rapid advance since the shaping of the term proteomics in 1997 (James, 1997).

#### 2.2.3. Protein-protein interactions

For studies of protein-protein interactions, the major methods used are Yeast Two-Hybrid (Y2H) screens, Affinity Purification Mass Spectrometry (AP-MS), or Bimolecular Fluorescence Complementation (BiFC) (reviewed in Zhang et al., 2010). Y2H assays take advantage of the bipartite structure of the yeast GAL4 transcriptional activator consisting of two functional domains, a transcription activation domain and a DNA-binding domain. In Y2H assays, the bait and the target protein are fused to the two functional domains of GAL4, respectively, together reconstituting the functional GAL4 protein that binds to its target promoter (UAS_GAL4_) to activate the expression of a down-stream gene encoding a selectable marker. Apart from a high false-positive rate, the use of yeast itself is a major drawback of the method. While cell type-specific cDNA libraries can be used to profile pairwise protein interactions, the system does not truly reflect the *in vivo* state of a specific plant cell (e.g., cofactors of an interaction may be missing). Several systems similar to Y2H assays have been established to specifically study membrane proteins (e.g., split-ubiquitin system; Obrdlik et al., 2004; Chen et al., 2010). For AP-MS, a bait protein is fused to an affinity tag for expression *in vivo*. The tagged protein of interest is subsequently purified as a complex with interacting proteins or other molecules and assayed by MS. This method is also associated with a relatively high false-positive rate due to protein contaminants. While the method is well-suitable for cell type-specific studies if the expression of the tagged protein is driven by a cell type-specific promoter, true omics-scale profiling can hardly be achieved, as a precondition would be the cell type-specific tagging of all proteins represented in a cell. This also holds true for BiFC, where a fluorescent protein (YFP, RFP, or GFP) is split into two non-fluorescent halves that are reconstituted to a fluorescent protein upon interaction of the bait and target proteins they are fused to (reviewed by Zhang et al., 2010). While BiFC has the advantage that spatial and temporal interactions can be resolved, it is also associated with a high false-positive rate. Consequently, methods for true cell type-specific large-scale protein-protein interaction studies in plants are lacking to date. Nonetheless, the currently available data on protein-protein interactions, as for example the recently established membrane protein interactome (Chen et al., 2012), may help to resolve certain dependencies within regulatory networks (see Sheth and Thaker, 2014, for a summary of the available databases).

#### 2.2.4. Protein-DNA interactions

Interactions between proteins and DNA comprises several functional aspects, for example nucleosome occupancy, specific histone modifications, or transcription factor binding. These interactions may be studied using either Chromatin ImmunoPrecipitation (ChIP, Orlando and Paro, 1993), or DNA adenine methyltransferase IDentification (Dam-ID, van Steensel and Henikoff, 2000). In both cases, the interaction of one protein (variant) with the DNA is monitored genome-wide. During the ChIP procedure, the DNA is cross-linked by formaldehyde to bound proteins before fragmentation by sonication. Chromatin fragments are then isolated with antibodies against the protein (variant) of interest. After recovery of the co-purified DNA by reverting the cross-links, the DNA sequence can be identified using microarray hybridization or high-throughput sequencing (He et al., 2011). Protocols facilitating cell type-specific ChIP (Chromatin Affinity purification from Specific cell Types by ChIP; CAST-ChIP), without the need for purification of the cell type of interest or a protein-specific antibody, have been developed (Schauer et al., 2013). However, these protocols rely on transgenics and specific promoters. In addition, we are not aware of a report where this method has been applied in plants or used to study rare cell types.

For Dam-ID, the protein of interest is fused to an adenine-methyltransferase of *E. coli* (Dam, Greil et al., 2006). Endogenous methylation of adenine is absent in most eukaryotes. Upon expression of the fusion protein, Dam is targeted to the native binding sites of the protein fused to it. This results in a localized methylation of adenines in the GATC sequence context. These regions can then be identified using methylation-sensitive restriction enzymes and microarray hybridization or high-throughput sequencing (Greil et al., 2006; Luo et al., 2011). Tissue or cell type-specific expression of the fusion protein can be used to overcome the need for cell isolation and has been shown to be highly specific (targeted DamID, “TaDa,” Southall et al., 2013). The major disadvantages of the method are the requirement for transgenics and specific promoters, as well as the need for optimization of the expression level to avoid untargeted methylation and toxicity of the Dam fusion protein. Thus, both approaches are currently quite laborious and generally only applicable to model-species. Nonetheless, especially transcription factor binding is of great value for the study of transcriptional networks (Yuan et al., 2008). If cell type-specific data is not available, previously identified transcription factor binding motifs may still help to identify transcriptional modules (Diez et al., 2014).

#### 2.2.5. Protein microarrays

Protein microarrays are a promising tool for proteomics as well as for interactions of proteins with other proteins, nucleic acids, cellular surface markers, or posttranslational protein-modifications (Yang et al., 2011b; Uzoma and Zhu, 2013). Several different types of protein microarrays can therefore be distinguished. On analytical microarrays, well characterized proteins (e.g., monoclonal antibodies) are spotted to identify a specific set of proteins. Alternatively, less well characterized proteins (e.g., lysates from whole cells) are spotted on functional microarrays to test for interaction partners. Finally, proteome microarrays hold the majority of encoded proteins for an organism (Yang et al., 2011b). While the first proteome microarray for budding yeast was established in 2001 (reviewed by Uzoma and Zhu, 2013), not many applications were reported in plants (Yang et al., 2011b; Uzoma and Zhu, 2013). Nevertheless, protein microarrays have, for example, successfully been used to study 802 transcription factors in *Arabidopsis* (almost half of all transcription factors annotated in *Arabidopsis*, Gong et al., 2008). While protein microarrays may have a high potential for applications in systems biology, they are currently still limited by high production costs and laborious production methods (e.g., large-scale cloning of open reading frames, protein purification, and production of high-affinity monoclonal antibodies, Yang et al., 2011b).

#### 2.2.6. Metabolomics

Due to the high complexity of plant metabolites coming from both primary and secondary metabolism, the plant metabolome is highly complex. Although by far not comprehensively elucidated to date, about 200,000 different metabolites are estimated to be represented in plants (reviewed by Sheth and Thaker, 2014). While a variety of analysis platforms can in principle be applied for metabolite detection, Nuclear Magnetic Resonance (NMR) and MS are the most frequently used methods (Kueger et al., 2012; Sheth and Thaker, 2014). High resolution mapping of metabolites has recently been achieved in *Arabidopsis* roots by combining FACS with high resolution MS (Moussaieff et al., 2013). In addition, glandular trichomes have been used as model systems for large-scale metabolome analyses (Tissier, 2012). However, the major limitation of current metabolomics is the lack of a single method allowing comprehensive measurements in terms of qualitative detection, quantitation, and spatio-temporal resolution. This is the case because the metabolites differ significantly in their concentration, chemical properties, and analytical behavior. Two major strategies in metabolome profiling are the use of either targeted or untargeted MS (reviewed in Kueger et al., 2012). Targeted MS relies on previous knowledge about structures and chemical properties of the metabolites of interest and combines chromatographic separation techniques, e.g., High Pressure Liquid Chromatography (HPLC) or Gas Chromatography (GC), with MS techniques. In contrast, non-targeted analyses using MS without prior chromatographic separation is used to profile metabolites without prior knowledge about their abundance or structure. This method often only allows the determination of metabolic signatures, as the characterization of a specific metabolite, for example by NMR, is highly challenging. Therefore, a key problem is the availability of reference spectra and compounds for compound identification and annotation (Kueger et al., 2012). Thus, the need for comprehensive databases including relevant information on the compounds, e.g., spectra, and the requirement for integration of metabolome data with other large-scale omics data has been noted (Fukushima et al., 2009). Current online resources include the Golm Metabolome Database (gmd.mpimp-golm.mpg.de) and the MASSBANK Database (www.massbank.jp).

An alternative method to study, for example, metabolites at spatial resolution without the need for prior cell isolation is MALDI-MS Imaging (MSI, reviewed by Lee et al., 2012). For MSI, a suitable matrix is directly applied to thin tissue sections (e.g., 10–20 μm). The prepared tissue sections are then rasterized with a laser-beam coupled to a high mass resolution (TOF-MS, reviewed in Kaspar et al., 2011). The spot size of the laser thereby determines the resolution. Only recently, technical improvements allowed to reach resolutions required for the analysis of single cells (< 20 μm, reviewed in Kueger et al., 2012; Lee et al., 2012). MSI has rarely been used in plants for proteomics, and only few studies reported the imaging of metabolites (reviewed in Kaspar et al., 2011; Kueger et al., 2012). Examples for metabolite imaging with MSI include the measurement of wheat grain cell-wall polysaccharides (Veličković et al., 2014, 100 μm spot size), or the lipid measurements in embryos of cotton (Horn et al., 2012, 35 μm spot size). While MSI has a great potential for cell type-specific studies for plant systems biology, it needs to be noted that only thin surface layers of < 1 μm are sampled by MALDI (Lee et al., 2012). However, further improvements in MSI are likely to be developed soon and adaption of these methods to plant tissues may once facilitate single-cell proteomics as well as metabolomics in a range of species.

In addition, to study the subcellular localization of specific ions or metabolites and their physiological relocation, e.g., by directed transport, a variety of molecular sensors has recently been developed. Such sensors usually depend on proteins changing their conformation upon binding of a specific substrate. Consequently, the distance between attached fluorescent proteins will change leading to an alteration in Fluorescent Resonance Energy Transfer (FRET, reviewed by Okumoto, 2012; Okumoto et al., 2012). For spatially and temporally resolved measurements, FRET can be measured by, for instance, Fluorescence Lifetime Imaging Microscopy (FLIM, reviewed by De Los Santos et al., 2015). While being very valuable tools in plant research, these techniques do not readily allow the high-throughput analysis of a large number of compounds in a plant cell and will thus not be discussed in detail in this review.

#### 2.2.7. DNA methylation

DNA (cytosine) methylation is a heritable epigenetic modification of the genome and is involved in various cellular and developmental processes in a wide range of species, including animals, fungi, and plants. Several methods for genome-wide profiling of the DNA cytosine methylation status have been established. These include the hybridization onto whole-genome DNA microarrays after digestion of genomic DNA with methylation-sensitive restriction enzymes, or the precipitation of methylated DNA with antibodies targeting methylated cytosines (Methylated DNA ImmunoPrecipitation, MeDIP), followed by either microarray hybridization (MeDIP-chip) or NGS (MeDIP-Seq, reviewed by Su et al., 2011; Ji et al., 2015). The current method of choice for methylome profiling is Whole-Genome Bisulfite Sequencing (WGBS). In brief, DNA is incubated with bisulfite, converting all unmethylated cytosines to uracils, which are identified as thymines during sequencing. In contrast, all methylated cytosines are protected from the conversion, remain unchanged, and are identified as cytosines during sequencing (Ji et al., 2015). Compared to the profiling of other epigenetic marks, such as histone modifications, WGBS has two major advantages. It does not require the use of transgenic plants or antibodies, and recently developed methods allow WGBS on as little as 125 pg of DNA (Post-Bisulfite Adaptor Tagging (PBAT), Miura et al. (2012); 20 pg diluted *Arabidopsis* DNA with a modified protocol, our unpublished data). WGBS is therefore a very promising method for the profiling of specific cell types in plants.

## 3. Systems biology approach toward plant development

As evident from the previous examples, plant cell type-specific systems biology is most advanced in cell types that can relatively easily be isolated in large enough amounts of suitable for any type of omics approach. For the root hairs of soybean, for example, a promising method to isolate large quantities facilitating any omics analysis has recently been described and will likely be of great use (Qiao and Libault, 2013). The method uses an ultrasound aeroponic system to enhance root hair density, followed by fixation and separation of the root hairs in liquid nitrogen. In addition, for the different cell types of the *Arabidopsis* root, FACS yields sufficient material for most omics approaches. An advantage of these systems is that due to the use of only one isolation method, the variability imposed by it can be held constant over all experiments. The use of a single method is also cost-efficient as it requires less time and resources to optimize only one method as compared to several. Due to the relatively easy sample collection and their physiological roles, roots, root hairs, and trichomes are excellent models to study responses to environmental stimuli, host-pathogen/symbiont interactions, metabolic pathways, or the dynamics of cellular specification and cell-cell communication in complex tissues. However, even the root may not be an optimal model to address fundamental questions of developmental systems biology. Its main disadvantages are the long developmental time span, starting very early during embryogenesis, and the complex interplay within and between the different cell types of the root, but also with the above-ground tissues, and biotic and abiotic environmental factors. Ideally, a developmental model system should allow an experimental coverage of the entire life-span of the organism. It would be of advantage if the organism were short-lived and comprise only a limited number of developmental stages and specialized cell and tissue types to reduce complexity and increase the affordability of comprehensive studies. For comparative analyses and evolutionary systems biology approaches, it would be further advantageous if the phylogeny of the model system included a broad range of organisms with gradual phenotypic changes, or with gain, loss, and alternative usage of modular building blocks. Finally, an ideal model system is most beneficial if its understanding can lead to direct applications in, for example, production of food or pharmaceuticals.

An intuitive model for the development of an organism is the embryo. During plant embryogenesis, the basic body organization with an apical-basal and radial pattern is established starting from a single cell, the zygote. The mature embryo already contains the progenitors of the main organizers of plant growth, the primary Shoot and Root Apical Meristems (SAM and RAM), and the hypocotyl and cotyledons with their various tissue types (reviewed in Lau et al., 2012). However, it is thus already a relatively complex system composed of multiple cell and tissue types. Additional complexity is imposed by the different stages of embryo development, spanning the time between the one-cellular zygote and the mature embryo. An in-depth systems biological description of embryogensis would therefore require sampling of a large variety of cell types at many time points. Nevertheless, while most transcriptional studies published so far focussed on whole tissues or entire embryos (reviewed in Palovaara et al., 2013; Zhan et al., 2015), recently, high-quality cell type-specific transcriptomes of the proembryo and the suspensor of the early stages of the *Arabidopsis* embryo were described (Slane et al., 2014).

Alternative models for the development of organisms, which are far less complex than the embryo, are the gametophytes of flowering plants: the pollen (male) and the embryo sac (female). They are typically formed from one spore (meiotic product) and, at maturity, they consist of only a few cells and cell types, including the male and female gametes, the sperm cells and the egg and central cells, respectively (reviewed in Yang et al., 2010; Twell, 2011; Schmidt et al., 2015). Upon double fertilization, the egg cell and the central cell fuse with one sperm each to give rise to the embryo and endosperm, respectively. The latter nurtures the embryo and acts as storage organ for seed reserves in many species, including the cereals. The endosperm is thus the most important food and feed source.

Given the sheer amount of pollen produced by a single plant, and the relatively simple isolation procedures for some of the specific cell types of the male germline in developing pollen, multiple cell type-specific transcriptome data sets are available from different species, including *Arabidopsis thaliana, Oryza sativa* (rice), *Zea mays* (maize), *Lilium longiflorum* (lily), and *Plumbago zeylanica* (white leadwort) (Table 1; reviewed in Schmidt et al., 2012; Anderson et al., 2013; Dukowic-Schulze et al., 2014; Kelliher and Walbot, 2014), and several cell type-specific proteomes have recently been described for tobacco, *Lilium davidii* var. unicolor (Lanzhou lily), and tomato (Table 1; Abiko et al., 2013; Chaturvedi et al., 2013; Zhao et al., 2013; Ischebeck et al., 2014). Due to its characteristic tip-growth, pollen tubes also serve as an excellent model to study cell elongation and mechanical properties of the cell wall (Vogler et al., 2013). However, pollen development is strikingly uniform in angiosperms (Maheshwari, 1950), and inter-species comparisons would therefore likely be more fruitful in gymnosperms, which show a remarkable variation in terms of the number of cell divisions between meiosis and the subsequent specification of the sperm cells (Fernando et al., 2010). In contrast to pollen, a plant forms much fewer female gametophytes, which are deeply embedded in the maternal floral tissue (e.g., in *Arabidopsis*, each flower contains around 50 ovules, each of which harbors only one embryo sac). Nonetheless, several cell type-specific transcriptomes (Table 2; reviewed in Schmidt et al., 2012; Wuest et al., 2013, and more recent data in Anderson et al., 2013; Okada et al., 2013; Schmidt et al., 2014) as well as a proteome analysis for rice egg cells (Table 2; Abiko et al., 2013) are currently available. Even though it is more difficult to collect than the pollen, the embryo sac has certain developmental features rendering it a highly interesting model system for plant development: (i) high evolutionary diversity within angiosperms, (ii) syncytial development (i.e., the formation of a multinucleate cell), (iii) specification and differentiation of only three to four distinct cell types, and (iv) a process in which plants can reproduce asexually via seeds (gametophytic apomixis).

**Table 1:** **Summary of transcriptome (top) and proteome (bottom) datasets generated for specific cell types during formation of the male reproductive lineage and gametogenesis**.

**Species**	**Cell type**	**Profiling method**	**Literatures**
*Oryza sativa* ssp. *Japonica*	Meiocyte	44K Agilent microarray	Tang et al., 2010
*Zea mays*	Meiocyte	RNA-Seq (Illumina)	Dukowic-Schulze et al., 2014
*Arabidopsis thaliana*	Meiocyte	RNA-Seq (SOLiD)	Yang et al., 2011a
*Arabidopsis thaliana*	Meiocyte	RNA-Seq (Illumina)	Chen et al., 2010
*Arabidopsis thaliana*	Meiocyte, UNM	CATMA microarray	Libeau et al., 2011
*Arabidopsis thaliana*	UNM	Affymetrix ATH1 microarray	Honys and Twell, 2004
*Oryza sativa* ssp. *Japonica*	UNM	Affymetrix rice genome array	Wei et al., 2010
*Lolium longiflorum*	GC	cDNA microarray	Okada et al., 2007
*Arabidopsis thaliana*	SC	Affymetrix ATH1 microarray	Borges et al., 2008
*Plumbago zeylanica*	SC	cDNA spotted microarray	Gou et al., 2009
*Oryza sativa* ssp. *Japonica*	SC	RNA-Seq (Illumina)	Anderson et al., 2013
*Nicotiana tabacum*	Meiocyte, tetrad UNM, polarized UNM	gel LC-MS	Ischebeck et al., 2014
*Solanum lycopersicum* (ecotype Red Setter)	Meiocyte, tetrad, UNM, polarized UNM	gel LC-Orbitrap-MS	Chaturvedi et al., 2013
*Lilium davidii* var. unicolor	SC, GC	MS/MS with MALDI-TOF/TOF	Zhao et al., 2013
*Oryza sativa* ssp. *Nipponbare*	SC	LC-MS/MS	Abiko et al., 2013

*In brief, pollen formation starts with a microspore mother cell (or meiocyte) which undergoes meiosis to give rise to a tetrad of reduced spores. Each of these microspores undergoes pollen mitosis I to give rise to a generative and a vegetative cell. The subsequent mitotic division of the generative cell (pollen mitosis II) results in the formation of two sperm cells (Twell, 2011). UNM, uninucleate microspore; GC, generative cell; SC, sperm cell; LC, liquid chromatography*.

**Table 2:** **Summary of transcriptome (top) and proteome (bottom) datasets generated for specific cell types during formation of the female reproductive lineage and gametogenesis**.

**Species**	**Cell type**	**Profiling method**	**Literatures**
*Arabidopsis thaliana*	MMC	ATH1 microarray	Schmidt et al., 2011
*Arabidopsis thaliana*	egg, cen, syn	ATH1 microarray	Wuest et al., 2010
*Arabidopsis thaliana*	cen	RNA-Seq (SOLiD)	Schmid et al., 2012
*Arabidopsis thaliana*	egg, syn	RNA-Seq (SOLiD)	Schmidt et al., 2014
*Oryza sativa* ssp. *Nipponbare*	egg, syn	44K Agilent microarray	Ohnishi et al., 2011
*Boechera gunnisoniana*	AIC, egg, cen, syn	ATH1 microarray, RNA-Seq (SOLiD)	Schmidt et al., 2014
*Hieracium praealtum*	AI	RNA-Seq (Roche 454)	Okada et al., 2013
*Oryza sativa* ssp. *Nipponbare*	egg	LC-MS/MS	Abiko et al., 2013

*MMC, megaspore mother cell; AIC, apomictic initial cell; AI, aposporous initial cell; egg, egg cell; syn, synergids; cen, central cell; LC, liquid chromatography*.

**Figure 2:**
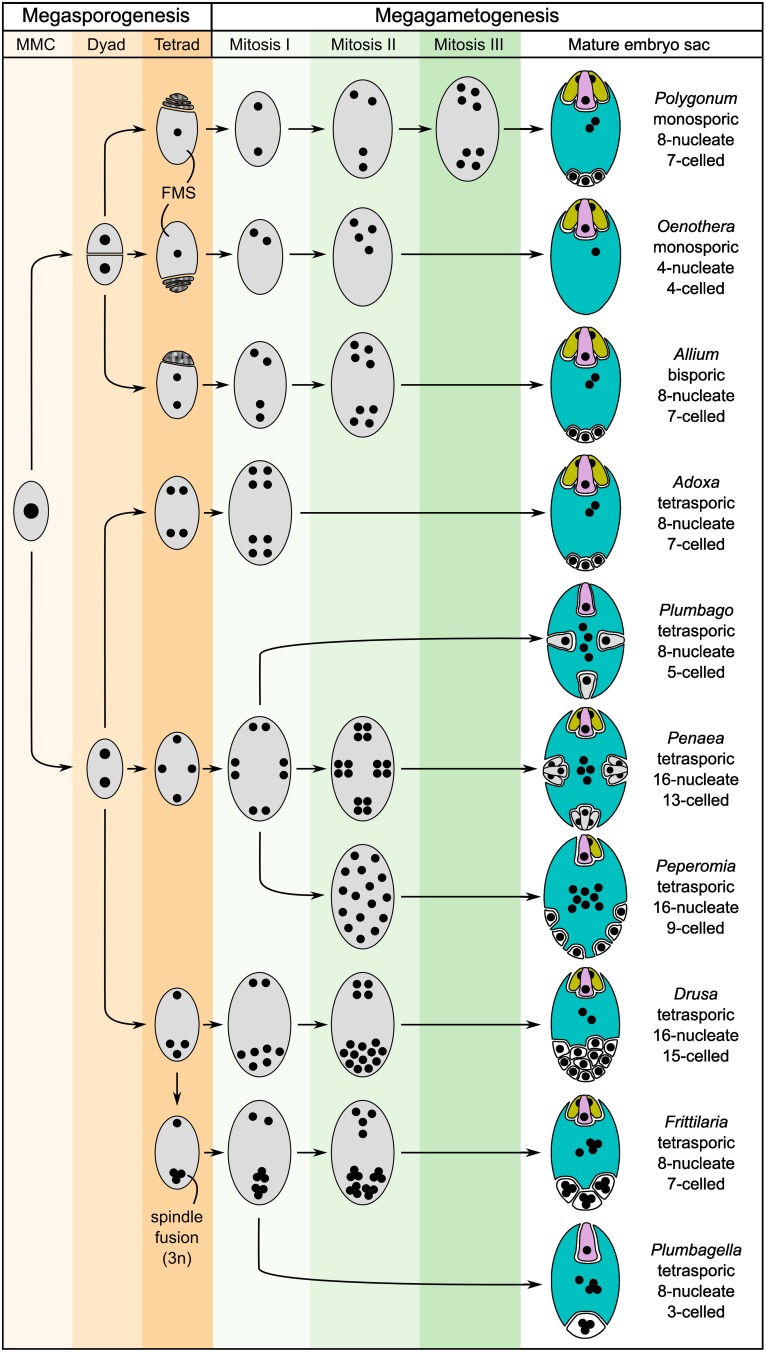
**Schematic showing several basic types of female gametophyte development in angiosperms and the structural diversity of the mature embryo sacs (after Maheshwari, 1950)**. The development of the female gametophyte can be devided into two steps: megasporogenesis (orange shading) and megagametogenesis (green shading). During megasporogenesis, a selected sporophytic cell, the megaspore mother cell (MMC), undergoes meiosis to give rise to spores. In most angiosperms, a tetrad of four megaspores is formed, of which three subsequently abort, leaving only one functional megaspore (FMS) to participate in megagametogenesis (e.g., *Polygonum*-type). However, a high diversity of the developmental processes of megasporogenesis and megagametogenesis has been observed in different genera, with variations, for example, including bispory and tetraspory. During megagametogenesis, the mature female gametophyte is formed through mitotic divisions, nuclear migration, and cellularization. For the mature embryo sac, the colors indicate the cell types: egg (pink), synergids (yellow), central cell (blue), and antipodal/lateral cells (white). Cells structurally similar to egg cells or synergids are drawn accordingly, but are colored gray.

The mature embryo sacs of angiosperms generally contain at least three distinct cell types: the synergids required for pollen tube attraction and reception, and the two gametes, the egg and the central cell. An exception are, for example, the *Podostemaceae*, where the central cell seems to degenerate before pollen tube arrival, resulting in a single fertilization event (Sehgal et al., 2014). In addition, antipodal cells are frequently present, but little is known about their function. It has been hypothesized that they might be involved in nutrient transfer from the surrounding tissues to the embryo sac (Raghavan, 1997). Despite the high functional similarity of mature embryo sacs, their formation is highly diverse across different plant taxa (Figure 2; Maheshwari, 1950; Huang and Russell, 1992; Baroux et al., 2002; Williams and Friedman, 2004). Reproductive development can be divided into two steps: megasporogenesis and megagametogenesis. Megasporogenesis comprises the formation and maturation of the initial meiotic products (megaspores) from a single selected sporophytic cell, the Megaspore Mother Cell (MMC), and is under the control of the usually diploid sporophytic genome. Megagametogenesis describes the following mitotic divisions, cellularization, cell specification, and maturation of the female gametophyte, which is under the control of the typically haploid genome. Both processes exhibit high diversity within angiosperms. Depending on the number of spores that survive and participate in megagametogenesis, megasporogenesis can be divided into *monosporic* (one spore), *bisporic* (two spores), and *tetrasporic* (all four spores). Further variation includes the location of the degenerating spores and the positioning of the spores in the *tetrasporic* types. Likewise, megagametogenesis can vary in the number of mitotic divisions, the arrangement of the nuclei/cells, and late divisions of individual cells after cellularization (e.g., in *Amborella*, Friedman, 2006). Comparative analysis of the structure of a wide range of embryo sacs and reconstruction of the ancestral state suggest that the embryo sacs of early angiosperms contained only four cells: two synergids, one egg cell and one central cell. It has been hypothesized that duplication of this four-celled module facilitated the emergence of the bi-nucleate central cell that, following fertilization, forms an endosperm with a maternal:paternal genome contribution ratio of 2:1 (Williams and Friedman, 2004; Friedman, 2006; Friedman and Ryerson, 2009). This unequal parental contribution to the endosperm has received a lot of attention over the last century. As a tissue protecting and nourishing the embryo, the endosperm may be subject to adaptive processes and parental conflicts (Haig and Westoby, 1989; Baroux et al., 2002).

An interesting aspect of female gametophyte development (and *tetrasporic* megasporogenesis) is the formation of a syncytium during the divisions of the nuclei prior to cellularization. In angiosperms, gametogenesis and early stages of endosperm development are the two major examples for the formation of a syncytium. In contrast, the plasmodial tapetum, for example, is formed by degeneration of the cell walls and the fusion of the resulting protoplasts (Furness and Rudall, 1998). Unlike regular cell divisions, where the positions of cells are relatively fixed due to the rigid cell wall, a syncytium allows for nuclear migration and for differentiation according to gradients of positional information. Indeed, determination of cell fate in the embryo sac of *Arabidopsis* depends on the position of the nuclei as, for example, indicated by the *Arabidopsis retinoblastoma-related1 (rbr1)* mutant, which produces supernumerary nuclei differentiating according to their position within the FG (Johnston et al., 2008; Sprunck and Groß-Hardt, 2011). However, the nature of such information is still under debate. Appealing candidates may be gradients of plant hormones, such as cytokinin or auxin. For both, a role in establishing polarity during embryo sac development has been proposed (reviewed in Schmidt et al., 2015) but their role may be rather indirect (Lituiev et al., 2013). However, an alternative or complementary hypothesis can be formulated using the analogy to the syncytial embryogenesis in *Drosophila*, where around 70% of the genes expressed during early embryogenesis show a specific subcellular localization of their mRNA in the syncytium. Interestingly, specific subcellular mRNA localization peaks around the transition from syncytial to cellular development, potentially reflecting the high demand for localization mechanisms (Lécuyer et al., 2007). Thus, a fascinating possibility is that the specific subcellular localization of mRNAs in the syncytial stage of the developing embryo sac may play a role in determining cell fate. A possibility to test this hypothesis would be to separately isolate specific subcellular regions (e.g., the two opposing poles) of the developing syncytial female gametophyte and to compare the transcriptional profiles of these regions with each other.

Another interesting variation of reproductive development is gametophytic apomixis. It refers to the process of asexual reproduction through seeds in the absence of fertilization (reviewed in Koltunow and Grossniklaus, 2003). Apomixis occurs in more than 400 plant species from around 40 genera and is likely of polyphyletic origin (Asker and Jerling, 1992; Carman, 1997). Gametophytic apomixis involves the omission or abortion of meiosis (apomeiosis) and the formation of an embryo from an unfertilized egg (parthenogenesis), while the endosperm can be formed by autonomous development of the central cell or dependent on fertilization (pseudogamy). Depending on the mechanism of the formation of the unreduced megaspore, the resulting offspring can be genetically completely identical to the mother plant without any chromosomal rearrangements. It is thereby possible to fix complex genotypes over multiple generations without a loss in heterozygosity. While gametophytic apomixis is absent in major crop plants, engineered apomictic crops would promise great potential and economical value for plant breeding and agriculture (Koltunow et al., 1995; Vielle-Calzada et al., 1996; Grossniklaus et al., 1998). From a developmental perspective, apomixis can be seen as an alteration of the sexual pathway, where certain processes are initiated too early or in the wrong cell type (Koltunow, 1993; Grossniklaus, 2001). Detailed understanding of the molecular processes and pathways governing gametogenesis during sexual and apomictic reproduction is therefore a precondition to engineer apomixis in crop plants. In evolutionary terms, apomixis is a highly interesting trait. On one hand, it allows the dispersal of seeds without the need for a sexual partner (Smith, 1978) and may therefore be advantageous for the colonization of new habitats (Tomlinson, 1966). On the other hand, the trade-off for this clonal reproduction appears to be very costly. Apomicts may accumulate deleterious mutations over many generations (Muller, 1964) and their populations are likely of low genetic variability, which reduces their potential to adapt to a changing environment. Recent proposals, however, suggest that epigenetic variation may also contribute to adaptive potential, which may explain the ecological success of many apomicts (Hirsch et al., 2012).

Given the natural variation in sexual and apomictic species, the female gametophyte of angiosperms can be seen as an excellent model system to study fundamental developmental processes and evolutionary aspects of plant development and biology that are of high importance to agriculture. Its simple organization and the relatively few developmental stages would allow for an in-depth analysis of various species enabling evolutionary comparisons at the whole-genome level. Given the high diversity, inter-species comparisons may identify genes and genetic networks involved in the emergence of evolutionary novelties, such as the unequal genetic contribution of the two parents to the endosperm or gametophytic apomixis. Deciphering the evolutionary mechanisms underlying these processes may also provide an answer to the long-standing question, how useful research on model organisms is for crop improvement. However, the small size and inaccessibility of the cell types of developing and mature embryo sacs make the isolation and subsequent application of omics methods very difficult. Aside the challenges associated with data integration and analysis, data generation is hence a major limiting factor. In general, the main obstacle with most approaches is the number of cells required for in-depth profiling of a certain molecule (e.g., protein or metabolite). This may be overcome by either increased sensitivity of the profiling method, or through a simplified collection of a large number of cells. However, most high-throughput isolation methods (e.g., for FACS/FANS/INTACT) rely on the existence of a specific marker (i.e., a cell type-specific promoter) and the possibility to generate transgenic plants. In addition, typically a certain abundance of the cell type of interest in the sample is required for efficient sorting and purification. Given that these preconditions are generally not met by low abundant cell types of of non-model organisms, it is likely that plant systems biology will profit the most from an increase in sensitivity and the development of novel profiling methods. In the following sections, we will therefore focus on a subset of omics approaches, which are readily available or which bear great future potential for routine large-scale *in vivo* profiling of specific cell types. The examples given are restricted to studies on specific cell types of the female gametophytes of angiosperms.

### 3.1. Transcriptome

Transcriptomics is clearly the most frequently used and currently the most robust omics approach to study female gametophyte and plant reproductive development. Following the early transcriptional profiling with low-throughput technologies [early Expressed Sequence Tag (EST) sequencing projects, reviewed in Wuest et al. (2013)], cell type-specific transcriptomes were generated for the egg cell, the central cell, the synergids, and the MMC of *Arabidopsis* (Wuest et al., 2010; Schmidt et al., 2011; Schmid et al., 2012), the egg cell and the synergids for rice (Ohnishi et al., 2011; Anderson et al., 2013), all cell types of the mature embryo sac and the Apomictic Initial Cell (AIC) of *Boechera gunnisoniana* (a close apomictic relative of *Arabidopsis thaliana* where an AIC is specified instead of a sexual MMC, Schmidt et al., 2014), and the AIC of *Hieracium praealtum* (hawkweed, where the AIC is formed by an additional sporophytic cell developing adjacent to the sexual reproductive lineage, Okada et al., 2013; Table 2). Given the requirement to establish a specific gene expression profile for cell specification and differentiation, transcriptomics is also especially suitable as a first approach toward an unknown species, because it provides a comprehensive snapshot of the cellular instruction machinery. It further enables the identification of cell type-specific markers and can thus provide a basis for other approaches, like detailed molecular and mechanistic studies. The advantage of transcriptional profiling as compared to proteomic studies is the possibility to amplify the material prior to detection. Several RNA-Seq protocols allow transcriptional profiling of single cells corresponding to as little as about 10 pg of total RNA (reviewed in Head et al., 2014). This low detection limit facilitates the use of relatively low throughput isolation methods, such as LAM or manual microdissection, allowing the profiling of specific cell types of embryo sacs in model and non-model species (Okada et al., 2013; Wuest et al., 2013; Schmidt et al., 2014). A current drawback of the amplification strategy is the introduction of potential quantification biases. A possible solution may be Unique Molecular Identifiers (UMI). These are short sequences with random nucleotides (e.g., 1024 different UMIs with 5 random nucleotides), which are used to label initial cDNA molecules prior to amplification. An excess of UMIs compared to the number of identical cDNAs ensures that each combination of a given UMI with a certain cDNA is unique. After amplification and sequencing, this can be used to differentiate between individual molecules in the initial cDNA pool and duplicates originating from cDNA amplification (i.e., to count molecules instead of reads, Islam et al., 2014). An interesting approach for future studies may be Fluorescent *In Situ* RNA SEQuencing (FISSEQ), in which stably cross-linked cDNA amplicons are sequenced directly within a biological sample, thereby not only quantifying gene expression, but also detecting the subcellular localization of the transcripts (Lee et al., 2014). Improvement of this method and its adaption to plant tissues would thus undoubtfully be a major advance in cell type-specific transcriptional profiling.

### 3.2. Proteome and metabolome

Proteomics and metabolomics on specific cell types is substantially more challenging than transcriptomics. A current limitation for cell type-specific proteomics is the large discrepancy between the number of detected proteins compared to the number of expressed genes, which is due to the low sensitivity of proteomics methods towards low-abundant proteins. An additional complexity arises by the presence of a wide range of post-translational modifications, such as phosphorylation or glycosylation. Apart from two early examples, identifying only the major proteins in the egg cells of maize and rice (6 and 4 proteins, Okamoto et al., 2004; Uchiumi et al., 2007), we are only aware of the recent description of the egg cell proteome in rice, where 2138 proteins were identified using around 500 egg cells (Abiko et al., 2013; Table 2). In the same study, 2179 proteins were identified starting from 30,000 isolated sperm cells (Table 1; Abiko et al., 2013). Given the further improvements of the sensitivity of mass spectrometers, the example demonstrates that proteomics of purified cells of the female gametopyhte should be possible for cases where enough material can be collected. Mechanical or manual isolation of female gametes was reported for a variety of species including barley, wheat, rape seed, maize, tobacco, *Torenia, Alstroemeria*, and *Arabidopsis* (Kranz et al., 1991; Holm et al., 1994; Kovács et al., 1994; Katoh et al., 1997; Tian and Russell, 1997; Sprunck et al., 2005; Hoshino et al., 2006; Okuda et al., 2009; Jullien et al., 2012). In most of these species, we anticipate that the protocols would already allow the isolation of sufficient material for MS-based proteomics. Another promising approach for future experiments may be MSI, circumventing the need for (laborious) cell purification.

### 3.3. Methylome

DNA cytosine methylation (5mC) plays an important role in the epigenetic regulation of plant genomes. While WGBS has not yet been reported for isolated cells of the female gametophyte, bisulfite sequencing of specific sequences has already been applied for *Arabidopsis* central cells and synergids isolated by LAM (Wöhrmann et al., 2012; You et al., 2012). It would likely be possible to combine LAM or manual microdissection with WBGS. This would thus allow methylome profiling of gametes in model as well as non-model species. Importantly, this may provide novel insights into the molecular basis underlying heterosis (Groszmann et al., 2011), characterized by superior characteristics of F1 hybrid plants as compared to their parents. While epigenetic regulatory pathways are likely important for heterosis, their precise involvement remains elusive to date (Chen, 2013). Understanding of the regulatory mechanisms governing heterosis is of great interest for plant breeding and crop production. Importantly, gametophytic development and early stages of embryogenesis are likely important for the establishment of heterosis.

## 4. Conclusion and perspectives

To date, cell type-specific systems biology in plants is frequently constrained by the difficulties associated with the isolation of the cell type of interest in large enough amounts. Robust and simple isolation methods exist only for a few cell types. Consequently, the comprehensive profiling of all cell types of an organism with different large-scale profiling methods, allowing the detailed understanding of all biological processes ongoing in the biological system, is still an unreached goal. While the in-depth understanding of complex organisms over their lifespan is a major aim for systems biology, the use of simple model organisms bears advantages, given the persisting technical limitations. We introduce the female gametophyte of angiosperms as an attractive model system for future systems biology approaches in plant development. Apart from its relatively simple organization, it is of great biological and agronomical importance, for example with respect to seed production and plant breeding.

Currently, most high-throughput isolation methods with broader application (e.g., FACS/FANS/INTACT) are limited to model organisms (e.g., *Arabidopsis thaliana, Oryza sativa*). However, a biological system may be best understood in the context of evolution. In addition, a detailed understanding of the cellular processes in major agriculturally important species including wheat, where an additional challenge is the genome size and its hexaploid nature, are a precondition for targeted crop improvement. Such studies would thus not only be of potential applied value, but would also help to understand the common concepts and divergent mechanisms active in different species. Therefore, methods facilitating large-scale profiling of specific cell types in model as well as non-model organism are of crucial importance. Parallel high-throughput profiling of several organisms covering a phenotypic gradient, or including gain, loss, and alternative usage of modular building blocks along the phylogeny, will enable evolutionary systems biology. This approach may ultimately help to reconstruct the emergence of evolutionary novelties and to find the underlying genetic and molecular networks. Such an understanding would in turn allow the control of the underlying processes with an unprecedented resolution. In perspective, this can be an important precondition for targeted improvement of crop species, including the engineering of apomixis into crop plants.

Even though the isolation of individual cell types is currently still very challenging, the rapid technical advances observed over the past few years in, for example, transcriptional profiling, are clear indications for the tremendous improvement of large-scale profiling technologies. In this light, we emphasize methods for transcriptomics, proteomics, metabolomics, and methylomics, in which we see great future potential. However, cell type-specificity and single-cell resolution are just one step towards a more comprehensive view on developmental processes and environmental responses. Clearly, monitoring subcellular localization of molecules and their interactions will be essential to understand certain patterning processes and specific cellular functions. In analogy to the hypothesized distribution of mRNA within the syncytial female gametophyte, subcellular localization of mRNA may also occur within the cell types of the mature female gametophyte to, for example, target the proteins they encode to a specific subcellular region. In this respect, technologies based on high resolution imaging, allowing large-scale profiling without prior cell isolation, for example MSI or FISSEQ, are very promising for future applications. The growing amount of data and data types also points to the need for novel computational solutions addressing the problems of data storage, integration, and analysis (see Ahrens et al., 2007; Yuan et al., 2008; Fukushima et al., 2009; Chuang et al., 2010; Katari et al., 2010; Liberman et al., 2012; Fukushima et al., 2014; Gomez-Cabrero et al., 2014; Robinson et al., 2014). The current situation, in which data sometimes remain unpublished, are frequently poorly annotated, and widely dispersed in specialized databases, may be taken as motivation to develop integrative computational platforms specifically focussing on future data. Considering the almost exponential growth of biological data over the last years (Ideker et al., 2001; Chuang et al., 2010), these platforms may also ignore data from the past to allow for innovative solutions. In this context, standardized data formats and annotation, easily accessible databases, powerful data mining tools, user-friendly and freely available software, as well as scalable storage platforms are the current and future demands in systems biology (Chuang et al., 2010; Gomez-Cabrero et al., 2014).

### Conflict of interest statement

The authors declare that the research was conducted in the absence of any commercial or financial relationships that could be construed as a potential conflict of interest.

## References

[B1] Abiko M. Furuta K. Yamauchi Y. Fujita C. Taoka M. Isobe T.. (2013). Identification of proteins enriched in rice egg or sperm cells by single-cell proteomics. PLoS ONE 8:e69578. 10.1371/journal.pone.0069578 23936051 PMC3723872

[B2] Ahrens C. H. Wagner U. Rehrauer H. K. Türker C. Schlapbach R. (2007). Current challenges and approaches for the synergistic use of systems biology data in the scientific community. Experientia Suppl. 97, 277–307. 10.1007/978-3-7643-7439-6/12 17432272

[B3] Anderson S. N. Johnson C. S. Jones D. S. Conrad L. J. Gou X. Russell S. D.. (2013). Transcriptomes of isolated *Oryza sativa* gametes characterized by deep sequencing: evidence for distinct sex-dependent chromatin and epigenetic states before fertilization. Plant J. 76, 729–741. 10.1111/tpj.12336 24215296

[B4] Asker S. E. Jerling L. (1992). Apomixis in Plants. London, UK: CRC Press.

[B5] Bailey-Serres J. (2013). Microgenomics: genome-scale, cell-specific monitoring of multiple gene regulation tiers. Annu. Rev. Plant Biol. 64, 293–325. 10.1146/annurev-arplant-050312-120035 23451787

[B6] Baroux C. Spillane C. Grossniklaus U. (2002). Evolutionary origins of the endosperm in flowering plants. Genome Biol. 3:reviews1026.1–reviews1026.5. 10.1186/gb-2002-3-9-reviews1026 PMC139410 12225592

[B7] Benfey P. N. (2012). Toward a systems analysis of the root. Cold Spring Harb. Symp. Quant. Biol. 77, 91–96. 10.1101/sqb.2012.77.014506 23234807 PMC3883509

[B8] Birnbaum K. Shasha D. E. Wang J. Y. Jung J. W. Lambert G. M. Galbraith D. W.. (2003). A gene expression map of the *Arabidopsis* root. Science 302, 1956–1960. 10.1126/science.1090022 14671301

[B9] Boone C. (2014). Yeast systems biology: our best shot at modeling a cell. Genetics 198, 435–437. 10.1534/genetics.114.169128 25316779 PMC4196597

[B10] Borges F. Gomes G. Gardner R. Moreno N. McCormick S. Feijó J. A.. (2008). Comparative transcriptomics of *Arabidopsis* sperm cells. Plant Physiol. 148, 1168–1181. 10.1104/pp.108.125229 18667720 PMC2556834

[B11] Bostein D. Fink G. R. (2011). Yeast: an experimental organism for 21st century biology. Genetics 189, 695–704. 10.1534/genetics.111.130765 22084421 PMC3213361

[B12] Brady S. M. Orlando D. A. Lee J. Y. Wang J. Y. Koch J. Dinneny J. R.. (2007). A high-resolution root spatiotemporal map reveals dominant expression patterns. Science 318, 801–806. 10.1126/science.1146265 17975066

[B13] Brechenmacher L. Lee J. Sachdev S. Song Z. Nguyen T. H. N. Joshi T.. (2009). Establishment of a protein reference map for soybean root hair cells. Plant Physiol. 149, 670–682. 10.1104/pp.108.131649 19036831 PMC2633823

[B14] Brechenmacher L. Lei Z. Libault M. Findley S. Sugawara M. J. Sadowsky M. J.. (2010). Soybean metabolites regulated in root hairs in response to the symbiotic bacterium *Bradyrhizobium japonicum*. Plant Physiol. 153, 1808–1822. 10.1104/pp.110.157800 20534735 PMC2923908

[B15] Brem R. B. Yvert G. Clinton R. Kruglyak L. (2002). Genetic dissection of transcriptional regulation in budding yeast. Science 296, 752–755. 10.1126/science.1069516 11923494

[B16] Brown J. A. Sherlock G. Myers C. L. Burrows N. M. Deng C. Wu H. I.. (2006). Global analysis of gene function in yeast by quantitative phenotypic profiling. Mol. Syst. Biol. 2, 2006.0001. 10.1038/msb4100043 16738548 PMC1681475

[B17] Carman J. G. (1997). Asynchronous expression of duplicate genes in angiosperms may cause apomixis, bispory, tetraspory, and polyembryony. Biol. J. Linnean Soc. 61, 51–94. 10.1111/j.1095-8312.1997.tb01778.x

[B18] Chaturvedi P. Ischebeck T. Egelhofer V. Lichtscheidl I. Weckwerth W. (2013). Cell-specific analysis of the tomato pollen proteome from pollen mother cell to mature pollen provides evidence for developmental priming. J. Proteome Res. 12, 4892–4903. 10.1021/pr400197p 23731163

[B19] Chen C. Farmer A. D. Langley R. J. Mudge J. Crow J. A. May G. D.. (2010). Meiosis-specific gene discovery in plants: RNA-Seq applied to isolated *Arabidopsis* male meiocytes. BMC Plant Biol. 10:280. 10.1186/1471-2229-10-280 21167045 PMC3018465

[B20] Chen J. Lalonde S. Obrdlik P. Noorani Vatani A. Pasra S. A. Vilarino C.. (2012). Uncovering *Arabidopsis* membrane protein interactome enriched in transporters using mating-based split ubiquitin assays and classification models. Front. Plant Sci. 3:124. 10.3389/fpls.2012.00124 22737156 PMC3380418

[B21] Chen Z. J. (2013). Genomic and epigenetic insights into the molecular bases of heterosis. Nat. Rev. Genet. 14, 471–482. 10.1038/nrg3503 23752794

[B22] Chuang H. Y. Hofree M. Ideker T. (2010). A decade of systems biology. Annu. Rev. Cell Dev. Biol. 26, 721–744. 10.1146/annurev-cellbio-100109-104122 20604711 PMC3371392

[B23] Costanzo M. Baryshnikova A. Bellay J. Kim Y. Spear E. D. Sevier C. S.. (2010). The genetic landscape of a cell. Science 327, 425–431. 10.1126/science.1180823 20093466 PMC5600254

[B24] Dai S. Chen S. (2012). Single-cell-type proteomics: toward a holistic understanding of plant function. Mol. Cell. Proteomics 11, 1622–1630. 10.1074/mcp.R112.021550 22982375 PMC3518137

[B25] Dai X. Wang G. Yang D. S. Tang Y. Broun P. Marks M. D.. (2010). TrichOME: a comparative omics database for plant trichomes. Plant Physiol. 152, 44–54. 10.1104/pp.109.145813 19939948 PMC2799345

[B26] De Los Santos C. Chang C. W. Mycek M. A. Cardullo R. A. (2015). FRAP, FLIM, and FRET: detection and analysis of cellular dynamics on a molecular scale using fluorescence microscopy. Mol. Reprod. Dev. 82, 587–604. 10.1002/mrd.22501 26010322 PMC4515154

[B27] Deal R. B. Henikoff S. (2011). The INTACT method for cell type-specific gene expression and chromatin profiling in *Arabidopsis thaliana*. Nat. Protoc. 6, 56–68. 10.1038/nprot.2010.175 21212783 PMC7219316

[B28] Diez D. Hutchins A. P. Miranda-Saavedra D. (2014). Systematic identification of transcriptional regulatory modules from protein-protein interaction networks. Nucleic Acids Res. 42:e6. 10.1093/nar/gkt913 24137002 PMC3874207

[B29] Dukowic-Schulze S. Sundararajan A. Mudge J. Ramaraj T. Farmer A. D. Wang M.. (2014). The transcriptome landscape of early maize meiosis. BMC Plant Biol. 14:118. 10.1186/1471-2229-14-118 24885405 PMC4032173

[B30] Evrard A. Bargmann B. O. Birnbaum K. D. Tester M. Baumann U. Johnson A. A. (2012). Fluorescence-activated cell sorting for analysis of cell type-specific responses to salinity stress in *Arabidopsis* and rice. Methods Mol. Biol. 913, 265–276. 10.1007/978-1-61779-986-0/18 22895766 PMC4164160

[B31] Fernando D. D. Quinn C. R. Brenner E. D. Owens J. N. (2010). Male gametophyte development and evolution in extant gymnosperms. Int. J. Plant Dev. Biol. 4, 47–63. Available online at: http://www.globalsciencebooks.info/JournalsSup/10IJPDB_4_SI1.html

[B32] Friedman W. E. (2006). Embryological evidence for developmental lability during early angiosperm evolution. Mol. Syst. Biol. 2, 20060001. 10.1038/nature04690 16710419

[B33] Friedman W. E. Ryerson K. C. (2009). Reconstructing the ancestral female gametophyte of angiosperms: insights from *Amborella* and other ancient lineages of flowering plants. Am. J. Bot. 96, 129–143. 10.3732/ajb.0800311 21628180

[B34] Fukushima A. Kanaya S. Nishida K. (2014). Integrated network analysis and effective tools in plant systems biology. Front. Plant Sci. 5:598. 10.3389/fpls.2014.00598 25408696 PMC4219401

[B35] Fukushima A. Kusano M. Redestig H. Arita M. Saito K. (2009). Integrated omics approaches in plant systems biology. Curr. Opin. Chem. Biol. 13, 532–538. 10.1016/j.cbpa.2009.09.022 19837627

[B36] Furness C. A. Rudall P. J. (1998). The tapetum and systematics in monocotyledons. Bot. Rev. 64, 201–239. 10.1007/BF02856565

[B37] Gomez-Cabrero D. Abugessaisa I. Maier D. Teschendorff A. Merkenschlager M. Gisel A.. (2014). Data integration in the era of omics: current and future challenges. BMC Syst. Biol. 8:I1. 10.1186/1752-0509-8-S2-I1 25032990 PMC4101704

[B38] Gong W. He K. Covington M. Dinesh-Kumar S. P. Snyder M. Harmer S. L.. (2008). The development of protein microarrays and their applications in DNA-protein and protein-protein interaction analyses of *Arabidopsis* transcription factors. Mol. Plant 1, 27–41. 10.1093/mp/ssm009 19802365 PMC2756181

[B39] Gou X. Yuan T. Wei X. Russell S. D. (2009). Gene expression in the dimorphic sperm cells of *Plumbago zeylanica*: transcript profiling, diversity, and relationship to cell type. Plant J. 60, 33–47. 10.1111/j.1365-313X.2009.03934.x 19500307

[B40] Greil F. Moorman C. Van Steensel B. (2006). DamID: mapping of *in vivo* protein-genome interactions using tethered DNA adenine methyltransferase. Methods Enzymol. 410, 342–359. 10.1016/S0076-6879(06)10016-6 16938559

[B41] Grobei M. A. Qeli E. Brunner E. Rehrauer H. Zhang R. Roschitzki B.. (2009). Deterministic protein inference for shotgun proteomics data provides new insights into *Arabidopsis* pollen development and function. Genome Res. 19, 1786–1800. 10.1101/gr.089060.108 19546170 PMC2765272

[B42] Grossniklaus U. (2001). From sexuality to apomeiosis: molecular and genetic approaches, in The Flowering of Apomixis: from Mechanisms to Genetic Engineering, eds Savidan Y. Carman J. G. Dresselhaus T. (Mexico, DF: CIMMYT, IRD, European Commission DG VI (FAIR)), 168–211.

[B43] Grossniklaus U. Koltunow A. van Lookeren Campagne M. (1998). A bright future for apomixis. Trends Plant Sci. 3, 415–416. 10.1016/S1360-1385(98)01338-7

[B44] Groszmann M. Greaves I. K. Albertyn Z. I. Scofield G. N. Peacock W. J. Dennis E. S. (2011). Changes in 24-nt siRNA levels in *Arabidopsis* hybrids suggest and epigenetic contribution to hybrid vigor. Proc. Natl. Acad. Sci. U.S.A. 108, 2617–2622. 10.1073/pnas.1019217108 21266545 PMC3038704

[B45] Haig D. Westoby M. (1989). Parent-specific gene expression and the triploid endosperm. Am. Nat. 134, 147–155. 10.1086/284971

[B46] He G. Elling A. A. Deng X. W. (2011). The epigenome and plant development. Annu. Rev. Plant Biol. 62, 411–435. 10.1146/annurev-arplant-042110-103806 21438682

[B47] Head S. R. Komori H. K. LaMere S. A. Whisenant T. Van Nieuwerburgh F. Salomon D. R.. (2014). Library construction for next-generation sequencing: overviews and challenges. Biotechniques 56, 61–77. 10.2144/000114133 24502796 PMC4351865

[B48] Hirsch S. Baumberger R. Grossniklaus U. (2012). Epigenetic variation, inheritance, and selection in plant populations. Cold Spring Harb. Symp. Quant. Biol. 77, 97–104. 10.1101/sqb.2013.77.014605 23619013

[B49] Hollenbeck T. P. Siuzdak G. Blackledge R. D. (1999). Electrospray and MALDI mass spectrometry in the identification of spermicides in criminal investigations. J. Forensic Sci. 44, 793–788. 10.1520/JFS14553J 10432613

[B50] Holm P. B. Knudsen S. Mouritzen P. Negri D. Olsen F. L. Roue C. (1994). Regeneration of fertile barley plants from mechanically isolated protoplasts of the fertilized egg cell. Plant Cell 6, 531–543. 10.1105/tpc.6.4.531 12244247 PMC160456

[B51] Honys D. Twell D. (2004). Transcriptome analysis of haploid male gametophyte development in *Arabidopsis*. Genome Biol. 5:R85. 10.1186/gb-2004-5-11-r85 15535861 PMC545776

[B52] Horn P. J. Korte A. R. Neogi P. B. Love E. Fuchs J. Strupat K.. (2012). Spatial mapping of lipids at cellular resolution in embryos of cotton. Plant Cell 24, 622–636. 10.1105/tpc.111.094581 22337917 PMC3315237

[B53] Hoshino Y. Murata N. Shinoda K. (2006). Isolation of individual egg cells and zygotes in *Alstroemeria* followed by manual selection with a microcapillary-connected micropump. Ann. Bot. 97, 1139–1144. 10.1093/aob/mcl072 16621859 PMC2803394

[B54] Hu T. X. Yu M. Zhao J. (2011). Techniques of cell type-specific transcriptome analysis and applications in researches of plant sexual reproduction. Front. Biol. 6, 31–39. 10.1007/s11515-011-1090-1

[B55] Huang B. Q. Russell S. D. (1992). Female germ unit: organization, isolation, and function. Int. Rev. Cytol. 140, 233–293. 10.1016/S0074-7696(08)61099-2

[B56] Ideker T. Galitski T. Hood L. (2001). A new approach to decoding life: systems biology. Annu. Rev. Genomics Hum. Genet. 2, 343–372. 10.1146/annurev.genom.2.1.343 11701654

[B57] Ischebeck T. Valledor L. Lyon D. Gingl S. Nagler M. Meijón M.. (2014). Comprehensive cell-specific protein analysis in early and late pollen development from diploid microsporocytes to pollen tube growth. Mol. Cell. Proteomics 13, 295–310. 10.1074/mcp.M113.028100 24078888 PMC3879621

[B58] Ishida T. Kurata T. Okada K. Wada T. (2008). A genetic regulatory network in the development of trichomes and root hairs. Annu. Rev. Plant Biol. 59, 365–386. 10.1146/annurev.arplant.59.032607.092949 18257710

[B59] Islam S. Zeisel A. Joost S. La Manno G. Zajac P. Kasper M.. (2014). Quantitative single-cell RNA-seq with unique molecular identifiers. Nat. Methods 11, 163–166. 10.1038/nmeth.2772 24363023

[B60] James P. (1997). Protein identification in the post-genome era: the rapid rise of proteomics. Q. Rev. Biophys. 30, 279–331. 10.1017/S0033583597003399 9634650

[B61] Ji L. Neumann D. A. Schmitz R. J. (2015). Crop epigenomics: identifying, unlocking, and harnessing cryptic variation in crop genomes. Mol. Plant 8, 860–870. 10.1016/j.molp.2015.01.021 25638564 PMC5121661

[B62] Johnston A. J. Matveeva E. Kirioukhova O. Grossniklaus U. Gruissem W. (2008). A dynamic reciprocal RBR-PRC2 regulatory circuit controls *Arabidopsis* gametophyte development. Curr. Biol. 18, 1680–1686. 10.1016/j.cub.2008.09.026 18976913

[B63] Jullien P. E. Susaki D. Yelagandula R. Higashiyama T. Berger F. (2012). DNA methylation dynamics during sexual reproduction in *Arabidopsis thaliana*. Curr. Biol. 22, 1825–1830. 10.1016/j.cub.2012.07.061 22940470

[B64] Kaspar S. Peukert M. Svatos A. Matros A. Mock H. P. (2011). MALDI-imaging mass spectrometry - an emerging technique in plant biology. Proteomics 11, 1840–1850. 10.1002/pmic.201000756 21462348

[B65] Katari M. S. Nowicki S. D. Aceituno F. F. Nero D. Kelfer J. Thompson L. P.. (2010). Virtual Plant: a software platform to support systems biology research. Plant Physiol. 152, 500–515. 10.1104/pp.109.147025 20007449 PMC2815851

[B66] Katoh N. Lörz H. Kranz E. (1997). Isolation of viable egg cells of rape (*Brassica napus* l). Zygote 5, 31–33. 10.1017/S0967199400003531 9223243

[B67] Kehr J. (2001). High resolution spation analysis of plant systems. Curr. Opin. Plant Biol. 4, 197–201. 10.1016/S1369-5266(00)00161-8 11312129

[B68] Kelliher T. Walbot V. (2012). Hypoxia triggers meiotic fate acquisition in maize. Science 337, 345–348. 10.1126/science.1220080 22822150 PMC4101383

[B69] Kelliher T. Walbot V. (2014). Maize germinal cell initials accommodate hypoxia and precociously express meiotic genes. Plant J. 77, 639–652. 10.1111/tpj.12414 24387628 PMC3928636

[B70] Kerk N. M. Ceserani T. Tausta S. L. Sussex I. M. Nelson T. M. (2003). Laser capture microdissection of cells from plant tissues. Plant Physiol. 132, 27–35. 10.1104/pp.102.018127 12746508 PMC1540312

[B71] Kitano H. (2002). Systems biology: a brief overview. Science 295, 1662–1664. 10.1126/science.1069492 11872829

[B72] Koltunow A. M. (1993). Apomixis: embryo sacs and embryos formed without meiosis or fertilization in ovules. Plant Cell 5, 1425–1437. 10.1105/tpc.5.10.1425 12271038 PMC160373

[B73] Koltunow A. M. Bicknell R. A. Chaudhury A. M. (1995). Apomixis: molecular strategies for the generation of genetically identical seeds without fertilization. Plant Physiol. 108, 1345–1352. 12228546 10.1104/pp.108.4.1345 PMC157511

[B74] Koltunow A. M. Grossniklaus U. (2003). Apomixis: a developmental perspective. Annu. Rev. Plant Biol. 54, 547–574. 10.1146/annurev.arplant.54.110901.160842 14503003

[B75] Konstantinidis K. T. Serres M. H. Romine M. F. Rodrigues J. L. M. Auchtung J. McCue L. A.. (2009). Comparative systems biology across an evolutionary gradient within the *Shewanella* genus. Proc. Natl. Acad. Sci. U.S.A. 106, 15909–15914. 10.1073/pnas.0902000106 19805231 PMC2747217

[B76] Kovács M. Barnabás B. Kranz E. (1994). The isolation of viable egg cells of wheat (*Triticum aestivum* L.). Sex. Plant Reprod. 7, 311–312. 10.1007/BF00227715

[B77] Kranz E. Bautor J. Lörz H. (1991). *In vitro* fertilization of single, isolated gametes of maize mediated by electrofusion. Sex. Plant Reprod. 4, 12–16. 10.1007/BF00194565

[B78] Kueger S. Steinhauser D. Willmitzer L. Giavalisco P. (2012). High-resolution plant metabolomics: from mass spectral features to metabolites and from whole-cell analysis to subcellular metabolite distributions. Plant J. 70, 39–50. 10.1111/j.1365-313X.2012.04902.x 22449042

[B79] Lau S. Slane D. Herud O. Kong J. Jürgens G. (2012). Early embryogenesis in flowering plants: setting up the basic body pattern. Annu. Rev. Plant Biol. 63, 483–506. 10.1146/annurev-arplant-042811-105507 22224452

[B80] Lécuyer E. Yoshida H. Parthasarathy N. Alm C. Babak T. Cerovina T.. (2007). Global analyses of mRNA localization reveals a prominent role in organizing cellular architecture and function. Cell 131, 174–187. 10.1016/j.cell.2007.08.003 17923096

[B81] Lee J. H. Daugharthy E. R. Scheiman J. Kalhor R. Yang J. L. Ferrante T. C.. (2014). Highly multiplexed subcellular RNA sequencing *in situ*. Science 343, 1360–1363. 10.1126/science.1250212 24578530 PMC4140943

[B82] Lee Y. J. Perdian D. C. Song Z. Yeung E. S. Nikolau B. J. (2012). Use of mass spectrometry for imaging metabolites in plants. Plant J. 70, 81–95. 10.1111/j.1365-313X.2012.04899.x 22449044

[B83] Libault M. Brechenmacher L. Cheng J. Xu D. Stacey G. (2010a). Root hair systems biology. Trends Plant Sci. 15, 641–650. 10.1016/j.tplants.2010.08.010 20851035

[B84] Libault M. Farmer A. Brechenmacher L. Drnevich J. Langley R. J. Bilgin D. D.. (2010b). Complete transcriptome of the soybean root hair cell, a single-cell model, and its alteration in response to *Bradyrhizobium japonicum* infection. Plant Physiol. 152, 541–552. 10.1104/pp.109.148379 19933387 PMC2815892

[B85] Libeau P. Durandet M. Granier F. Marquis C. Berthomé R. Renou J. P.. (2011). Gene expression profiling of *Arabidopsis* meiocytes. Plant Biol. 13, 784–793. 10.1111/j.1438-8677.2010.00435.x 21815983

[B86] Liberman L. A. Sozzani R. Benfey P. N. (2012). Integrative systems biology: an attempt to describe a simple weed. Curr. Opin. Plant Biol. 15, 162–167. 10.1016/j.pbi.2012.01.004 22277598 PMC3435099

[B87] Lituiev D. S. Krohn N. G. Müller B. Jackson D. Hellriegel B. Dresselhaus T.. (2013). Theoretical and experimental evidence indicates that there is no detectable auxin gradient in the angiosperm female gametophyte. Development 140, 4544–4553. 10.1242/dev.098301 24194471

[B88] Luo S. D. Shi G. W. Baker B. S. (2011). Direct targets of the *D. melanogaster* DSX^F^ protein and the evolution of sexual development. Development 138, 2761–2771. 10.1242/dev.065227 21652649 PMC3109601

[B89] Maheshwari P. (1950). An Introduction to the Embryology of Angiosperms. New York, NY: McGraw-Hill.

[B90] Mardis E. R. (2013). Next-generation sequencing platforms. Annu. Rev. Anal. Chem. 6, 287–303. 10.1146/annurev-anchem-062012-092628 23560931

[B91] Miura F. Enomoto Y. Dairiki R. Ito T. (2012). Amplification-free whole-genome bisulfite sequencing by post-bisulfite adaptor tagging. Nucleic Acids Res. 40:e136. 10.1093/nar/gks454 22649061 PMC3458524

[B92] Moussaieff A. Rogachev I. Brodsky L. Malitsky S. Toal T. W. Belcher H.. (2013). High-resolution metabolic mapping of cell types in plant roots. Proc. Natl. Acad. Sci. U.S.A. 110, E1232–E1241. 10.1073/pnas.1302019110 23476065 PMC3612672

[B93] Muller H. J. (1964). The relation of recombination of mutational advance. Mutat. Res. 106, 2–9. 10.1016/0027-5107(64)90047-8 14195748

[B94] Nestler J. Schütz W. Hochholdinger F. (2011). Conserved and unique features of the maize (*Zea mays* L) root hair proteome. J Proteome Res. 10, 2525–2537. 10.1021/pr200003k 21417484

[B95] Obrdlik P. El-Bakkoury M. Hamacher T. Cappellaro C. Vilarino C. Fleischer C.. (2004). K^+^ channel interactions detected by a genetic system optimized for systematic studies of membrane protein interactions. Proc. Natl. Acad. Sci. U.S.A. 101, 12242–12247. 10.1073/pnas.0404467101 15299147 PMC514463

[B96] Ohnishi T. Takanashi H. Mogi M. Takahashi H. Kikuchi S. Yano K.. (2011). Distinct gene expression profiles in egg and synergid cells of rice as revealed by cell type-specific microarrays. Plant Physiol. 155, 881–891. 10.1104/pp.110.167502 21106719 PMC3032473

[B97] Okada T. Hu Y. Tucker M. R. Taylor J. M. Johnson S. D. Spriggs A.. (2013). Enlarging cells initiating apomixis in *Hieractium praealtum* transition to an embryo sac program prior to entering mitosis. Plant Physiol. 163, 216–231. 10.1104/pp.113.219485 23864557 PMC3762643

[B98] Okada T. Singh M. B. Bhalla P. L. (2007). Transcriptome profiling of *Lilium longiflorum* generative cells by cDNA microarray. Plant Cell Rep. 26, 1045–1052. 10.1007/s00299-006-0300-9 17245599

[B99] Okamoto T. Higuchi K. Shinkawa T. Isobe T. Lörz H. Koshiba T.. (2004). Identification of major proteins in maize egg cells. Plant Cell Physiol. 45, 1406–1412. 10.1093/pcp/pch161 15564524

[B100] Okuda S. Tsutsui H. Shiina K. Sprunck S. Takeuchi H. Yui R.. (2009). Defensin-like polypeptide LUREs are pollen tube attractants secreted from synergid cells. Nature 458, 357–361. 10.1038/nature07882 19295610

[B101] Okumoto S. (2012). Quantitative imaging using genetically encoded sensors for small molecules in plants. Plant J. 70, 108–117. 10.1111/j.1365-313X.2012.04910.x 22449046

[B102] Okumoto S. Jones A. Frommer W. B. (2012). Quantitative imaging with fluorescent biosensors. Annu. Rev. Plant Biol. 63, 663–706. 10.1146/annurev-arplant-042110-103745 22404462

[B103] Orlando V. Paro R. (1993). Mapping *Polycomb*-repressed domains in the *bithorax* complex using *in vivo* formaldehyde cross-linked chromatin. Cell 75, 1187–1198. 10.1016/0092-8674(93)90328-N 7903220

[B104] Österlund T. Nookaew I. Nielsen J. (2012). Fifteen years of large scale metabolic modeling of yeast: developments and impacts. Biotechnol. Adv. 30, 979–988. 10.1016/j.biotechadv.2011.07.021 21846501

[B105] Palovaara J. Saiga S. Weijers D. (2013). Transcriptomics approaches in the early *Arabidopsis* embryo. Trends Plant Sci. 18, 514–521. 10.1016/j.tplants.2013.04.011 23726727

[B106] Pelkmans L. (2012). Using cell-to-cell variability – a new era in molecular biology. Science 336, 425–426. 10.1126/science.1222161 22539709

[B107] Perry R. H. Cooks R. G. Noll R. J. (2008). Orbitrap mass spectrometry: instrumentation, ion motion and applications. Mass Spectrom. Rev. 27, 661–699. 10.1002/mas.20186 18683895

[B108] Petricka J. J. Schauer M. A. Megraw M. Breakfield N. W. Thompson J. W. Georgiev S.. (2012). The protein expression landscape of the *Arabidopsis* root. Proc. Natl. Acad. Sci. U.S.A. 109, 6811–6818. 10.1073/pnas.1202546109 22447775 PMC3344980

[B109] Qiao Z. Libault M. (2013). Unleashing the potential of the root hair cell as a single plant cell type model in root systems biology. Front. Plant Sci. 4:484. 10.3389/fpls.2013.00484 24324480 PMC3840615

[B110] Raghavan V. (1997). Molecular Embryology of Flowering Plants. Cambridge, UK: Cambridge University Press.

[B111] Robinson S. W. Fernandes M. Husi H. (2014). Current advances in systems and integrative biology. Comput. Struct. Biotechnol. J. 11, 35–46. 10.1016/j.csbj.2014.08.007 25379142 PMC4212281

[B112] Rogers E. D. Jackson T. Moussaieff A. Aharoni A. Benfey P. N. (2012). Cell type-specific transcriptional profiling: implications for metabolite profiling. Plant J. 70, 5–17. 10.1111/j.1365-313X.2012.04888.x 22449039 PMC3315153

[B113] Sakata K. Komatsu S. (2014). Plant proteomics: from genome sequencing to proteome databases and repositories. Methods Mol. Biol. 1072, 29–42. 10.1007/978-1-62703-631-3/3 24136512

[B114] Schauer T. Schwalie P. C. Handley A. Margulies C. E. Flicek P. Ladurner A. G. (2013). CAST-ChIP maps cell-type-specific chromatin states in the *Drosophila* central nervous system. Cell Rep. 5, 271–282. 10.1016/j.celrep.2013.09.001 24095734 PMC5877786

[B115] Schilmiller A. L. Miner D. P. Larson M. McDowell E. Gang D. R. Wilkerson C.. (2010). Studies of a biochemical factory: tomato trichome deep expressed sequence tag sequencing and proteomics. Plant Physiol. 153, 1212–1223. 10.1104/pp.110.157214 20431087 PMC2899918

[B116] Schmid M. Davison T. S. Henz S. R. Pape U. J. Demar M. Vingron M.. (2005). A gene expression map of *Arabidopsis thaliana* development. Nat. Genet. 37, 501–506. 10.1038/ng1543 15806101

[B117] Schmid M. W. Grossniklaus U. (2015). Rcount: simple and flexible RNA-Seq read counting. Bioinformatics 31, 436–437. 10.1093/bioinformatics/btu680 25322836

[B118] Schmid M. W. Schmidt A. Klostermeier U. C. Barann M. Rosenstiel P. Grossniklaus U. (2012). A powerful method for transcriptional profiling of specific cell types in eukaryotes: laser-assisted microdissection and RNA sequencing. PLoS ONE 7:e29685. 10.1371/journal.pone.0029685 22291893 PMC3266888

[B119] Schmidt A. Schmid M. W. Grossniklaus U. (2012). Analysis of plant germline development by high-throughput RNA profiling: technical advances and new insights. Plant J. 70, 18–29. 10.1111/j.1365-313X.2012.04897.x 22449040

[B120] Schmidt A. Schmid M. W. Grossniklaus U. (2015). Plant germline formation: molecular insights define common concepts and illustrate developmental flexibility in apomictic and sexual reproduction. Development 142, 229–241. 10.1242/dev.102103 25564620

[B121] Schmidt A. Schmid M. W. Klostermeier U. C. Qi W. Guthörl D. Sailer C.. (2014). Apomictic and sexual germline development differ with respect to cell cycle, transcriptional, hormonal and epigenetic regulation. PLoS Genet. 10:e1004476. 10.1371/journal.pgen.1004476 25010342 PMC4091798

[B122] Schmidt A. Wuest S. E. Vijverberg K. Baroux C. Kleen D. Grossniklaus U. (2011). Transcriptome analysis of the *Arabidopsis* megaspore mother cell uncovers the importance of RNA helicases for plant germ line development. PLoS Biol. 9:e1001155. 10.1371/journal.pbio.1001155 21949639 PMC3176755

[B123] Schulze W. X. Usadel B. (2010). Quantitation in mass-spectrometry-based proteomics. Annu. Rev. Plant Biol. 61, 491–516. 10.1146/annurev-arplant-042809-112132 20192741

[B124] Sehgal A. Mann N. Mohan Ram H. Y. (2014). Structural and developmental variability in the female gametophyte of *Griffithella hookeriana, Polypleurum stylosum*, and *Zeylanidium lichenoides* and its bearing on the occurrence of single fertilization in Podostemaceae. Plant Reprod. 27, 205–223. 10.1007/s00497-014-0252-0 25394544

[B125] Sheth B. P. Thaker V. S. (2014). Plant systems biology: insights, advandes and challenges. Planta 240, 33–54. 10.1007/s00425-014-2059-5 24671625

[B126] Slane D. Kong J. Berendzen K. W. Kilian J. Henschen A. Kolb M.. (2014). Cell type-specific transcriptome analysis in the early *Arabidopsis thaliana* embryo. Development 141, 4831–4840. 10.1242/dev.116459 25411212

[B127] Smith J. M. (1978). The Evolution of Sex. Cambridge, UK: Cambridge University Press.

[B128] Southall T. D. Gold K. S. Egger B. Davidson C. M. Caygill E. E. Marshall O. J.. (2013). Cell-type-specific profiling of gene expression and chromatin binding without cell isolation: assaying RNA pol II occupancy in neural stem cells. Dev. Cell 26, 101–112. 10.1016/j.devcel.2013.05.020 23792147 PMC3714590

[B129] Soyer O. S. (2012). Evolutionary Systems Biology, Advances in Experimental Medicine and Biology, Vol. 751 Heidelberg: Springer.

[B130] Sprunck S. Baumann U. Edwards K. Langridge P. Dresselhaus T. (2005). The transcript composition of egg cells changes significantly following fertilization in wheat (*Triticum aestivum* L.). Plant J. 41, 660–672. 10.1111/j.1365-313X.2005.02332.x 15703054

[B131] Sprunck S. Groß-Hardt R. (2011). Nuclear behavior, cell polarity, and cell specification in the female gametophyte. Sex. Plant Reprod. 24, 123–136. 10.1007/s00497-011-0161-4 21336612

[B132] Su Z. Han L. Zhao Z. (2011). Conservation and divergence of DNA methylation in eukaryotes: new insights from single base-resolution DNA methylomes. Epigenetics 6, 134–140. 10.4161/epi.6.2.13875 20962593 PMC3278781

[B133] Süel G. M. Kulkarni R. P. Dworkin J. Garcia-Ojalvo J. Elowitz M. B. (2007). Tunability and noise depence in differentiation dynamics. Science 315, 1716–1719. 10.1126/science.1137455 17379809

[B134] Tang X. Zhang Z. Y. Zhang W. J. Zhao X. M. Li X. Zhang D.. (2010). Global gene profiling of laser-captured pollen mother cells indicates molecular pathways and gene subfamilies involved in rice meiosis. Plant Physiol. 154, 1855–1870. 10.1104/pp.110.161661 20959420 PMC2996036

[B135] Taylor-Teeples M. Ron M. Brady S. M. (2011). Novel biological insights revealed from cell type-specific expression profiling. Curr. Opin. Plant Biol. 14, 1–7. 10.1016/j.pbi.2011.05.007 21704550

[B136] Tian H. Q. Russell S. D. (1997). Micromanipulation of male and female gametes of *Nicotiana tabacum*: I. isolation of gametes. Plant Cell Rep. 16, 555–560. 10.1007/s002990050278 30727578

[B137] Tissier A. (2012). Glandular trichomes: what comes after expressed sequence tags? Plant J. 70, 51–68. 10.1111/j.1365-313X.2012.04913.x 22449043

[B138] Tomlinson J. (1966). The advantages of hermaphroditism and parthenogenesis. J. Theor. Biol. 11, 54–58. 10.1016/0022-5193(66)90038-5 5961543

[B139] Tucker M. R. Okada T. Hu Y. Scholefield A. Taylor J. M. Koltunow A. M. (2012). Somatic small RNA pathways promote the mitotic events of megagametogenensis during female reproductive development in *Arabidopsis*. Development 139, 1399–1404. 10.1242/dev.075390 22399683

[B140] Twell D. (2011). Male gametogenesis and germline specification in flowering plants. Sex. Plant Reprod. 24, 149–160. 10.1007/s00497-010-0157-5 21103996

[B141] Uchiumi T. Shinkawa T. Isobe T. Okamoto T. (2007). Identification of the major protein components of rice egg cells. J. Plant Res. 120, 575–579. 10.1007/s10265-007-0095-y 17558543

[B142] Uzoma I. Zhu H. (2013). Interactome mapping: using protein microarray technology to reconstruct diverse protein networks. Genomics Proteomics Bioinform. 11, 18–28. 10.1016/j.gpb.2012.12.005 23395178 PMC3968920

[B143] Van Cutsem E. Simonart G. Degand H. Faber A. M. Morsomme P. Boutry M. (2011). Gel-based and gel-free proteomic analysis of *Nicotiana tabacum* trichomes identifies proteins involved in secondary metabolism and in the (a)biotic stress response. Proteomics 11, 440–454. 10.1002/pmic.201000356 21268273

[B144] van Steensel B. Henikoff S. (2000). Identification of *in vivo* DNA targets of chromatin proteins using tethered Dam methyltransferase. Nat. Biotechnol. 18, 424–428. 10.1038/74487 10748524

[B145] Veličković D. Ropartz D. Guillon F. Saulnier L. Rogniaux H. (2014). New insights into the structural and spatial variability of cell-wall polysaccharides during wheat grain development, as revealed through MALDI mass spectrometry imaging. J. Exp. Bot. 65, 2079–2091. 10.1093/jxb/eru065 24600018 PMC3991742

[B146] Vielle-Calzada J. P. Crane C. Stelly D. M. (1996). Apomixis – the asexual revolution. Science 274, 1322–1323. 10.1126/science.274.5291.1322

[B147] Vogler H. Draeger C. Weber A. Felekis D. Eichenberger C. Routier-Kierzkowsky A. L.. (2013). The pollen tube: a soft shell with a hard core. Plant J. 73, 617–627. 10.1111/tpj.12061 23106269

[B148] Weckwerth W. (2011). Green systems biology – from single genomes, proteomes and metabolomes to ecosystems research and biotechnology. J. Proteomics 75, 284–305. 10.1016/j.jprot.2011.07.010 21802534

[B149] Wei L. Q. Xu W. Y. Deng Z. Y. Su Z. Xue Y. Wang T. (2010). Genome-scale analysis and comparison of gene expression profiles in developing and germinated pollen in *Oryza sativa*. BMC Genomics 11:338. 10.1186/1471-2164-11-338 20507633 PMC2895629

[B150] Weinhofer I. Hehenberger E. Roszak P. Hennig L. Köhler C. (2010). H3K27me3 profiling of the endosperm implies exclusion of *Polycomb* group protein targeting by DNA methylation. PLoS Genet. 6:e1001152. 10.1371/journal.pgen.1001152 20949070 PMC2951372

[B151] Weinhofer I. Köhler C. (2014). Endosperm-specific chromatin profiling by fluorescence-activated nuclei sorting and ChIP-on-chip. Methods Mol. Biol. 1112, 105–115. 10.1007/978-1-62703-773-0/7 24478010

[B152] Williams J. H. Friedman W. E. (2004). The four-celled female gametophyte of *Illicium* (Illiciaceae; Austrobaileyales): implications for understanding the origin and early evolution of monocots, eumagnoliids, and eudicots. Am. J. Bot. 91, 332–351. 10.3732/ajb.91.3.332 21653390

[B153] Wöhrmann H. J. P. Gagliardini V. Raissig M. T. Wehrle W. Arand J. Schmidt A.. (2012). Identification of a DNA methylation-independent imprinting control region at the *Arabidopsis MEDEA* locus. Genes Dev. 26, 1837–1850. 10.1101/gad.195123.112 22855791 PMC3426762

[B154] Wuest S. E. Grossniklaus U. (2014). Laser-assisted microdissection applied to floral tissues. Methods Mol. Biol. 1110, 329–344. 10.1007/978-1-4614-9408-9/19 24395268

[B155] Wuest S. E. Schmid M. W. Grossniklaus U. (2013). Cell-specific expression profiling of rare cell types as exemplified by its impact on our understanding of female gametophyte development. Curr. Opin. Plant Biol. 16, 41–49. 10.1016/j.pbi.2012.12.001 23276786

[B156] Wuest S. E. Vijverberg K. Schmidt A. Weiss M. Gheyselinck J. Lohr M.. (2010). *Arabidopsis* female gametophyte gene expression map reveals similarities between plant and animal gametes. Curr. Biol. 20, 506–512. 10.1016/j.cub.2010.01.051 20226671

[B157] Yang H. Lu P. Wang Y. Ma H. (2011a). The transcriptome landscape of *Arabidopsis* male meiocytes from high-throughput sequencing: the complexity and evolution of the meiotic process. Plant J. 65, 503–516. 10.1111/j.1365-313X.2010.04439.x 21208307

[B158] Yang L. Guo S. Li Y. Zhou S. Tao S. (2011b). Protein microarrays for systems biology. Acta Biochim. Biophys. Sin. 43, 161–171. 10.1093/abbs/gmq127 21257623 PMC7117600

[B159] Yang W. C. Shi D. Q. Chen Y. H. (2010). Female gametophyte development in flowering plants. Annu. Rev. Plant Biol. 61, 89–108. 10.1146/annurev-arplant-042809-112203 20192738

[B160] You W. Tyczewska A. Spencer M. Daxinger L. Schmid M. W. Grossniklaus U.. (2012). Atypical DNA methylation of genes encoding cysteine-rich peptides in *Arabidopsis thaliana*. BMC Plant Biol. 12:51. 10.1186/1471-2229-12-51 22512782 PMC3422182

[B161] Yuan J. S. Galbraith D. W. Dai S. Y. Griffin P. Stewart C. N. (2008). Plant systems biology comes of age. Trends Plant Sci. 13, 165–171. 10.1016/j.tplants.2008.02.003 18329321

[B162] Zhan J. Thakare D. Ma C. Lloyd A. Nixon N. M. Arakaki A. M.. (2015). RNA sequencing of laser-capture microdissected compartments of the maize kernel identifies regulatory modules associated with endosperm cell differentiation. Plant Cell 27, 513–531. 10.1105/tpc.114.135657 25783031 PMC4558669

[B163] Zhang Y. Gao P. Yuan J. S. (2010). Plant protein-protein interaction network and interactome. Curr. Genomics 11, 40–46. 10.2174/138920210790218016 20808522 PMC2851115

[B164] Zhao X. Yang N. Wang T. (2013). Comparative proteomic analysis of generative and sperm cells reveals molecular characteristics associated with sperm development and function specialization. J. Proteome Res. 12, 5058–5071. 10.1021/pr400291p 23879389

[B165] Zhu M. Dai S. McClung S. Yan X. Chen S. (2009). Functional differentiation of *Brassica napus* guard cells and mesophyll cells revealed by comparative proteomics. Mol. Cell. Proteomics 8, 752–766. 10.1074/mcp.M800343-MCP200 19106087 PMC2667361

